# MiRKAT-MC: A Distance-Based Microbiome Kernel Association Test With Multi-Categorical Outcomes

**DOI:** 10.3389/fgene.2022.841764

**Published:** 2022-04-01

**Authors:** Zhiwen Jiang, Mengyu He, Jun Chen, Ni Zhao, Xiang Zhan

**Affiliations:** ^1^ Department of Biostatistics, Gillings School of Global Public Health, University of North Carolina, Chapel Hill, NC, United States; ^2^ Department Department of Biostatistics and Bioinformatics, Rollins School of Public Health, Emory University, Atlanta, GA, United States; ^3^ Department of Quantitative Health Sciences, Mayo Clinic, Rochester, MN, United States; ^4^ Department of Biostatistics, Bloomberg School of Public Health, Johns Hopkins University, Baltimore, MD, United States; ^5^ Department of Biostatistics, School of Public Health and Beijing International Center for Mathematical Research, Peking University, Beijing, China

**Keywords:** beta-diversity, longitudinal studies, microbiome association analysis, multi-categorical outcomes, kernel association test

## Abstract

Increasing evidence has elucidated that the microbiome plays a critical role in many human diseases. Apart from continuous and binary traits that measure the extent or presence of a disease, multi-categorical outcomes including variations/subtypes of a disease or ordinal levels of disease severity are commonly seen in clinical studies. On top of that, studies with clustered design (i.e., family-based and longitudinal studies) are popular alternatives to population-based ones as they are able to identify characteristics on both individual and population levels and to investigate the trajectory of traits of interest over time. However, existing methods for microbiome association analysis are inadequate to handle multi-categorical outcomes, neither independent nor clustered data. We propose a microbiome kernel association test with multi-categorical outcomes (MiRKAT-MC). Our method is versatile to deal with both nominal and ordinal outcomes for independent and clustered data. In addition, it incorporates multiple ecological distances to allow for different association patterns between outcomes and microbiome compositions to be incorporated. A computationally efficient pseudo-permutation strategy is used to evaluate the statistical significance. Comprehensive simulations show that MiRKAT-MC preserves the nominal type I error and increases statistical powers under various scenarios and data types. We also apply MiRKAT-MC to real data sets with nominal and ordinal outcomes to gain biological insights. MiRKAT-MC is easy to implement, and freely available via an R package at https://github.com/Zhiwen-Owen-Jiang/MiRKATMC with a Graphical User Interface through R Shinny also available.

## 1 Introduction

The diverse microbial cells including bacteria, archaea, and fungi that colonize the mucosal and skin environment constitute the human microbiome (Gilbert et al., 2018). It is broadly acknowledged that the human microbiome and its interaction with the immune, endocrine, and nervous systems are associated with a variety of illnesses, ranging from inflammatory bowel disease (Ni et al., 2017), to cancer (Kostic et al., 2013a), and to major depressive disorder (Jiang et al., 2015). A key step in investigating the relationship between microbiome and human disorders lies in quantifying the taxonomic composition. Currently, the most commonly used method is through the sequencing of the 16S ribosomal RNA gene, which, as a biomarker, is present in all prokaryotic cells and reflects the evolutionary distance between distinct genomes. Computationally, the 16S rRNA sequencing tags can be assigned into Operational Taxonomic Units (OTU) or Amplicon Sequence Variants (ASV) as computational surrogate of microbial taxa (Schloss, 2010; Callahan et al., 2016). Through sequencing, the microbial community can be directly quantified, without the need of labor-intensive bacterial culturing. For instance, the disparity between microbiome communities from two samples can be assessed via an ecological distance/dissimilarity metric, such as the UniFrac distance (Lozupone and Knight, 2005) and the Bray-Curtis dissimilarity (Bray and Curtis, 1957).

Identifying links between microbiome and diseases is often achieved by microbiome-wide association studies (MWAS) (Kostic et al., 2013b), which in turn provide insight into the biological mechanisms of human health and disease conditions. The data type of the investigated outcomes varies from study to study. Typically, samples can be dichotomized as cases and controls when exploring human diseases. For example, (Naseribafrouei et al., 2014) discovered potential correlation between depression and fecal microbiota, where study participants were classified as depression vs. non-depression. On the other hand, multi-categorical (nominal or ordinal) outcomes are also frequently encountered and investigated in many microbiome studies. For instance, Scher et al. (Scher et al., 2013) explored the association between rheumatoid arthritis (RA) and gut microbiota by recruiting patients with three different categories of arthritis: new-onset RA, treated RA, and psoriatic arthritis (PsA). Parikh et al. (Parikh et al., 2020) investigated the association between Apolipoprotein E (APOE) alleles and gut microbiome in murine models, where the APOE gene encodes a major cholesterol carrier protein that supports lipid transport and injury repair in the brain. Polymorphism in APOE gene is a major risk for developing Alzheimer disease. In this study, the APOE gene was coded as a nominal variable of different genotypes (APOE2 APOE3, and APOE4). Furthermore, Schirmer et al. Schirmer et al. (Schirmer et al., 2018) investigated the association between severity of ulcerative colitis and gut microbiome, where disease severity was treated as an ordinal variable with four levels: inactive, mild, moderate and severe.

Association analysis between a host trait and microbiome compositions can be generally addressed by PERMANOVA (Anderson, 2001), which partitions the total variation across the microbiome data cloud in the space of a chosen dissimilarity measure into multiple directions. PERMANOVA is able to accommodate both binary and multi-categorical outcomes, but fails to incorporate multiple distance metrics, where distinct distances capture distinct underlying association patterns and therefore are more powerful under different circumstances. Hence, Tang et al. (Tang et al., 2016) proposed PERMANOVA-S to incorporate multiple distance metrics into a single test. However, it is not adequate to multi-categorical outcomes unless we combine multiple categories into a binary variable, which potentially leads to significant power loss. An alternative to PERMANOVA is the family of microbiome regression-based kernel association tests (MiRKAT) (Zhao et al., 2015; Wilson et al., 2021). Utilizing the classic mixed effect models, the MiRKAT approaches summarize the microbiome structure as a kernel similarity matrix (constructed through the sample-sample distance metric) and model it as a random effect. Adjusting for covariates is straightforward in this framework. The association test is conducted via a variance component score test with *p*-value calculated in multiple ways, including analytical (Chen et al., 2016; Zhan et al., 2017a), permutation (Koh et al., 2019) and fast pseudo-permutation approaches (Zhan et al., 2017b). However, existing MiRKAT tests are not able to accommodate multi-categorical outcomes.

Beyond population-based studies in which all samples are independent, nowadays, researchers frequently collect microbiome data that are clustered or longitudinal in nature. For instance, Goodrich et al. (Goodrich et al., 2014) collected stool samples from female twins in the United Kingdom to investigate the relationship between obesity and gut microbiome. Flores et al. (Flores et al., 2014) explored the effect of antibiotic use on temporal variability of the microbiome diversity and community structure in gut, palm and tongue. Methods available to address correlated outcomes in microbiome studies burgeoned in the recent years (Chen and Li, 2016; Zhan et al., 2018; Zhang et al., 2018; Koh et al., 2019). For instance, GLMM-MiRKAT (Koh et al., 2019) extends MiRKAT for continuous, binary and count outcomes in longitudinal studies. It adopts kernel regression-based generalized linear mixed models to construct variance component tests and uses permutations to calculate the *p*-value. Unfortunately, only exchangeable clusters which contain identical number of observations and the same time points can be permuted in this approach. Thus, the permutation procedure will be very complicated and inefficient for unbalanced study designs. On top of that, permutation tends to be computationally intensive when the sample size increases (especially for studies with multi-categorical outcomes) or when small *p*-values are needed for multiple comparison adjustment. These drawbacks also exist for PERMANOVA.

In this paper, we propose a new distance-based microbiome kernel association test for multi-categorical outcomes (MiRKAT-MC), when samples are independent or clustered. MiRKAT-MC works for both nominal and ordinal outcomes, through the use of the generalized logit model (GLM) and the proportional odds model (POM), respectively. We utilize a fast pseudo-permutation technique (Zhan et al., 2017b) to calculate *p*-values. This approach features several advantages over its potential competitors: 1) it avoids the complication in designing a suitable permutation scheme for inference; 2) it is computationally efficient and much faster than direct permutations; 3) it controls the type I error and maintains high statistical power compared to the analytical approach. For the last point, due to the small sample size and the over-dispersion in microbiome data, it is quite difficult to approximate the MiRKAT test statistics, especially for clustered/longitudinal data and for outcomes that are not normally distributed.

Another common challenge in distance-based methods lies in how to select an appropriate ecological distance to construct the kernel, because the statistical power highly depends on a proper kernel to capture the underlying association pattern. Attempting multiple kernels and cherry-picking the smallest *p*-value yields inflated type I errors. On the other hand, naively adjusting the results by Bonferroni correction will reduce the statistical power substantially, mainly because the individual tests are highly correlated. We propose an omnibus test that combines the individual *p*-values from tests with different kernels through the harmonic mean procedure (HMP) (Wilson, 2019). The omnibus test is not necessarily the most powerful one: which test is the most powerful depends on the true nature of association, which is unknown prior to analysis. Nevertheless, our omnibus test is robust regardless of the real association pattern in that it loses little power compared to the most powerful one, and is much more powerful than choosing an inappropriate kernel.

In summary, the goal of this paper is to introduce novel statistical methods to examine the association between a multi-categorical outcome (both nominal and ordinal) and microbiome composition under different study designs (e.g., independent design, clustered design). Our major contributions are two-fold. First, we have cast the association analysis between a multi-categorical outcome and microbiome composition into frameworks of generalized logit models and proportional odds models (with additional random effects accounting for within-cluster correlations for clustered design). Our second contribution is proposing a robust *p*-value calculation procedure via a novel fast pseudo-permutation technique (Zhan et al., 2017b), avoiding the complicated and time-consuming permutation approach yet providing valid statistical inference. Finally, we provide a free R software to implement our proposed methods. It is a useful tool for microbiome researchers to investigate the relationship between the microbiome community and a multi-categorical outcome under a wide range of study designs, which was not readily available before.

## 2 Materials and Methods

To associate microbiome compositions with a multi-categorical outcome, we build upon generalized logit models (GLM) for nominal outcomes and proportional odds models (POM) for ordinal outcomes, and relate the microbiome profile with the outcome through the flexible semi-parametric kernel machine regression framework (Zhao et al., 2015). Our proposed MiRKAT-MC includes MiRKAT-MCN (for nominal outcomes) and MiRKAT-MCO (for ordinal outcomes). For both tests, we propose two versions, one for independent samples and another for clustered/longitudinal samples through the use of additional random effects in the generalized logit mixed model (GLMM) or the proportional odds mixed model (POMM).

### 2.1 GLM and POM for Independent Data

We first describe the GLM and POM model without considering the high dimensional microbiome data. Let ***Y***_*i*_ denote the multi-categorical outcome with total *J* categories for the *i*-th subject. Here, *bmY*_*i*_ is a vector with the *j*-th element being *y*_*ji*_, a binary variable denoting whether the *i*-th sample belongs to the *j*-th category, *i* = 1, … , *N*, *j* = 1, … , *J*. That is, *y*_*ji*_ = 1 means subject *i* is of category *j* and otherwise, *y*_*ji*_ = 0. In practice, *y*_*ji*_ can represent any mutually-exclusive categorical traits (nominal and ordinal), such as subtypes of cancers and increasing levels of disease severity that $\sum_{j=1}^{J}y_{ji}=1$ . From a probability perspective, ***Y***_*i*_ can be considered as from a multinomial distribution with *J* categories. Let *π*_*j*_ (***x***_*i*_) = Pr (*y*_*ji*_ = 1| ***x***_*i*_) be the conditional probability that subject *i* is of category *j* with *∑*_*j*_ *π*_*j*_ (***x***_*i*_) = 1, where ***x***_*i*_ denotes the set of covariates that we want to associate *Y*_*i*_ with (such as race, gender and age). If *bmY*_*i*_ is nominal, we can set the last category *J* as a reference without loss of generalization, and form the following GLM:

$$\log\frac{\pi_{j}\left(x_{i}\right)}{\pi_{J}\left(x_{i}\right)}=\alpha_{j}+\beta_{j}^{\prime }x_{i},$$ (1)

where *j* = 1, … , *J* − 1. The left-hand side of Eq. 1 is the logit of a conditional probability, and each coordinate of ***β***_*j*_ represents the increase in log-odds of falling into category *j* vs. the reference category *J* resulting from a one-unit increase in the corresponding covariate while holding the other covariates constant. This model simultaneously describes the effects of ***x***_*i*_ on all outcome categories in contrast to the reference. In this model, parameters ***β***_*j*_, *j* = 1, … , *J* − 1 can be different among categories. If the categories are ordinal, we can utilize the order information and form the following POM:

$$\text{logit}\left(\nu_{j}\left(x_{i}\right)\right)=\log\frac{\nu_{j}\left(x_{i}\right)}{1-\nu_{j}\left(x_{i}\right)}=\alpha_{j}+\beta^{\prime }x_{i},$$ (2)

where *j* = 1, … , *J* − 1, and

$$\nu_{j}\left(x_{i}\right)=\overset{j}{\underset{h=1}{\sum }}\mathrm{Pr}\left(y_{hi}=1|x_{i}\right)=\pi_{1}\left(x_{i}\right)+\cdots +\pi_{j}\left(x_{i}\right).$$

Here, *ν*_*j*_ (***x***_*i*_) is the conditional cumulative probability, and the corresponding response, defined by ${\tilde{y}}_{ji}=\sum_{h=1}^{j}y_{hi}$ , is called the cumulative response. The ordinal information is thus utilized in the way that the original categories enter the groups in a sequence. In contrast to GLM, ***β*** here keeps constant across *J* − 1 logits and the intercepts have to satisfy *α*_1_ < … < *α*_*J*−1_ in the proportional odds model.

Finally, we notice that there are other recent attempts to develop association analysis for multi-categorical outcomes using multinomial logistic regression (i.e., GLM model (1)), usually in the context of genome wide association studies (He et al., 2021; Liu et al., 2021). Despite the shared motivations, MiRKAT-MC is distinct from existing methods in multiple aspects. First, none of the existing approaches specifically models ordinal outcomes and thus MiKAT-MC under POM is statistically novel. Second, MiRKAT-MC includes options that utilize GLMM and POMM (described Section 2.2) to accommodate non-independent data from more complicated study designs. Last, our pseudo-permutation approach for obtaining *p*-values is novel and tends to outperform the asymptotic results as in existing methods when sample sizes are small, which is usually the case in microbiome data.

### 2.2 GLMM and POMM for Clustered/Longitudinal Data

Similarly, we first describe the GLMM and POMM models without considering the complex microbiome data. Suppose cluster *i* has *m*_*i*_ observations. Let $Y_{ik}={\left(y_{1ik},\ldots ,y_{Jik}\right)}^{\prime }$ represent the multi-categorical outcome of the *k*-th observation in cluster *i*, *i* = 1, … , *n*, *k* = 1, … , *m*_*i*_ and $N=\sum_{i=1}^{n}m_{i}$ be the total number of observations in the study. Following notations in the previous section, let *π*_*j*_ (***x***_*ik*_| ***b***_*ji*_) = Pr (*y*_*jik*_ = 1| ***x***_*ik*_, ***b***_*ji*_) and setting the *J*-th category as reference, the GLMM for clustered/longitudinal data can be written as:

$$\log\frac{\pi_{j}\left(x_{ik}|b_{ji}\right)}{\pi_{J}\left(x_{ik}|b_{ji}\right)}=\alpha_{j}+x_{ik}^{\prime }\beta_{j}+u_{ik}^{\prime }b_{ji},$$ (3)

where $x_{ik}={\left(x_{ik1},\ldots ,x_{ikq}\right)}^{\prime }$ denote covariates and $\beta_{j}={\left(\beta_{j1},\ldots ,\beta_{jq}\right)}^{\prime }$ are corresponding regression coefficients, ***u***_*ik*_ is the design matrix for the random effect term ***b***_*ji*_. We introduce ***b***_*ji*_ to model correlations among observations within cluster *i* of category *j*. The model definition is completed by specifying the distribution of the random effect $b_{ji}∼N\left(0,G_{j}\right)$ , where the variance-covariance matrix ***G***_*j*_ for the *j*-th category is unstructured. We also allow ***b***_*ji*_ to be correlated across categories.

The corresponding POMM for ordinal outcomes is as follows:

$$\text{logit}\left(\nu_{j}\left(x_{ik}|b_{i}\right)\right)=\alpha_{j}+x_{ik}^{\prime }\beta +u_{ik}^{\prime }b_{i}.$$ (4)

One main difference between models (Eqs. 3, 4) lies in model (Eq. 4) restricts ***b***_*i*_ to be identical across category comparisions, and thus $b_{i}∼N\left(0,G\right)$ with a fixed variance-covariance matrix ***G***. Here, we essentially assume that the random effects across the ordered categories are the same, which guarantees in proportional odds. Specifically, for a fixed cluster *i*, the random effect ***b***_*i*_ has identical value across different categories *j*. But for different clusters *i* and *i*′, ***b***_*i*_ and ***b***_*i*′_ may be different and both have normal distribution N(0,G) . The variance-covariance matrix ***G*** is unstructured as well. The same constraints for ***α***_*j*_ and ***β*** as in model (Eq. 2) also apply in the POMM model (Eq. 4).

### 2.3 Microbiome Association Analysis Under Models for Multi-Categorical Variables

We extend the previous described models to incorporate the complex microbiome data. For independent data, let $z_{i}={\left(z_{i1},\ldots ,z_{ip}\right)}^{\prime }$ be the composition of *p* OTUs for sample *i* (subject to appropriate normalization and transformation). We relate the multivariate outcome to the microbiome community and the covariates with the following model

$$\eta_{ji}=\alpha_{j}+x_{i}^{\prime }\beta_{j}+h_{j}\left(z_{i}\right),$$ (5)

for *i* = 1, … , *N*, *j* = 1, … , *J*, where *η* = *g* (⋅) and *g* (⋅) is a link function. For GLM, *g* (*π*_*ji*_) = log (*π*_*ji*_/*π*_*Ji*_), *π*_*ji*_ = *E* (*y*_*ji*_|*h*_*ji*_), and *h*_*ji*_ = *h*_*j*_ (***z***_*i*_); for POM, *g* (*ν*_*ji*_) = log{*ν*_*ji*_/(1 − *ν*_*ji*_)}, $\nu_{ji}=E\left({\tilde{y}}_{ji}|h_{ji}\right)$ is the conditional mean of the cumulative response ${\tilde{y}}_{ji}$ . *h*_*j*_ (⋅) are unknown real functions corresponding to the effects of microbiome on the *j*-th category. For POM, *h*_*j*_ (⋅) are identical across categories, and *α*_*j*_ and ***β***_*j*_ are subject to the constraints described in model (Eq. 2).

For clustered studies, let *y*_*jik*_ be a binary variable denoting whether the *k*-th observation of the *i*-th cluster belongs to the *j*-th category, where *k* = 1, … , *m*_*i*_, *i* = 1, … , *n* and *j* = 1, … , *J*. We let $N=\sum_{i=1}^{n}m_{i}$ be the total number of observations. $z_{ik}={\left(z_{ik1},\ldots ,z_{ikp}\right)}^{\prime }$ represent *p* OTUs for the *k*-th observation in the *i*-th cluster. The mixed effect model proceeds as

$$\eta_{jik}=\alpha_{j}+x_{ik}^{\prime }\beta_{j}+u_{ik}^{\prime }b_{ji}+h_{j}\left(z_{ik}\right),$$ (6)

where *η*_*jik*_ = *g* [*E* (*y*_*jik*_| ***b***_*ji*_, *h*_*jik*_)], *h*_*jik*_ = *h*_*j*_ (***z***_*ik*_), and *g* (⋅) is the same link function as model (Eq. 5). To illustrate our methodology, we here give some specific examples of the random effects ***u***_*ik*_. When *u*_*ik*_ = 1, *b*_*ji*_ is the random intercept which can be assumed normally distributed $∼N\left(0,g_{jj}\right)$ . When $u_{ik}={\left(1,t_{ik}\right)}^{\prime }$ , where *t*_*ik*_ is the time for the *k*-th observation in the *i*-th cluster (for longitudinal studies), $b_{ji}={\left(b_{ji1},b_{ji2}\right)}^{\prime }$ denote the random intercept and random slope with a bivariate normal distribution $N\left(0,G_{jj}\right)$ , where $G_{jj}=\left(\begin{array}{cc} g_{jj11} & g_{jj12}\\ g_{jj21} & g_{jj22} \end{array}\right)$ . Usually, ***G***_*jj*_ is specified as “unstructured” in generalized linear mixed effect models, providing much flexibility to capture cluster specific correlations. Again, for POMM, *α*_*j*_, ***β***_*jm*_, and ***b***_*ji*_ are subject to the constraints described in model (Eq. 4), and *h*_*jik*_ (⋅) should be identical across categories.

Our primary goal is to test the null hypothesis *H*_0_: *h*_1_ (⋅) = … = *h*_*J*−1_ (⋅) = 0 in Eq. 5, 6. One feasible approach is to develop such a test leveraging the kernel machine regression-based association analysis framework (Zhao et al., 2015). Through the critical connection between kernel machine regression and mixed models (Liu et al., 2007), $h={\left(h_{1},\ldots ,h_{J-1}\right)}^{\prime }$ can be considered as random effect with mean **0** and variance ***K*** *. We assume that each $h_{j}={\left(h_{j1},\ldots ,h_{jN}\right)}^{\prime }$ for independent data (or $h_{j}={\left(h_{j11},\ldots ,h_{j1m_{1}},h_{j21},\ldots ,h_{jnm_{n}}\right)}^{\prime }$ for clustered data) is independent and is of the same (multivariate) distribution. In such a case, ***K*** * = ***I***_*J*−1_ ⊗ *τ* ***K***, where ***I***_*J*−1_ denote (*J* − 1)-th order identity matrix, *τ* is an unspecified constant, ***K*** is an *N* × *N* kernel matrix, and ⊗ denotes Kronecker product. Following (Zhao et al., 2015), the kernel matrix can be easily constructed by a specific ecological distance matrix ***D***

$$K=-\frac{1}{2}\left(I_{N}-\frac{1_{N}1_{N}^{\prime }}{N}\right)D^{2}\left(I_{N}-\frac{1_{N}1_{N}^{\prime }}{N}\right),$$ (7)

where **1**_*N*_ is a vector of 1’s and ***I***_*N*_ is the identity matrix.

Typical distance measures for microbiome data include the Bray-Curtis dissimilarity, the weighted, unweighted or generalized UniFrac distances (Lozupone and Knight, 2005). The kernel matrix defined by Eq. 7 measures sample-pairwise similarities. Using this transformation, ecological information (e.g., taxonomic or the phylogenetic relationship between taxa) encoded in the distance ***D*** is preserved in ***K***, and thus in the functions of microbiome effect *h*_*j*_ (⋅)’s (which are assumed to be in the space spanned by ***K***). As demonstrated in previous studies, the embedding of such ecological information may boost statistical power for detecting an underlying association under many scenarios (Zhao et al., 2015). Here, we first focus the simpler case in which a single distance (e.g., Bray-Curtis dissimilarity) is considered. Omnibus test utilizing multiple kernels will be described later in this session.

To develop the distance-based kernel association test, we further translate association analysis working model (Eqs. 5, 6) into matrix language. For independent data,

η=Xβ+h, (8)

where $\eta ={\left(\eta_{11},\eta_{12},\ldots ,\eta_{1N},\ldots ,\eta_{J-1,1},\ldots ,\eta_{J-1,N}\right)}^{\prime }$ , $X=I_{J-1}⊗\left[\begin{array}{cc} 1 & x_{1}^{\prime }\\ \vdots & \vdots \\ 1 & x_{N}^{\prime } \end{array}\right]$ , $\beta ={\left(\alpha_{1},\beta_{1}^{\prime },\ldots ,\alpha_{J-1},\beta_{J-1}^{\prime }\right)}^{\prime }$ , $h={\left(h_{11},h_{12},\ldots ,h_{1N},\ldots ,h_{J-1,1},\ldots ,h_{J-1,N}\right)}^{\prime }$ is distributed as multivariate normal with mean zero and covariance matrix ***K*** * = ***I***_*J*−1_ ⊗ *τ* ***K***. Hence, testing *H*_0_: ***h*** = **0** is equivalent to testing *H*_0_: *τ* = 0, which can be accomplished by a variance component score test. The mathematical derivation of the variance component score test can be found in Supplementary Section 1.1 of the online Supplementary Material. In brief, the test statistic for ***h*** = **0** in (Eq. 8) is

$$Q_{1}={\left(y^{*}-X\hat{\beta }\right)}^{\prime }WK^{*}W\left(y^{*}-X\hat{\beta }\right),$$ (9)

where ***y*** * is a working response vector, ***W*** is a working weight matrix, and $\hat{\beta }$ is the estimated coefficients under the null. For GLM, $y^{*}=D_{\pi }\left(y-\hat{\pi }\right)+X\hat{\beta }$ , where ***D***_*π*_ = *∂* ***η***/*∂* ***π***, $\hat{\pi }$ is a vector of fitted values returned by the null model ***η*** = ***Xβ***. $W={\left(D_{\pi }V_{\pi }D_{\pi }\right)}^{-1}$ and ***V***_*π*_ is the variance-covariance matrix of the multinomial distribution evaluated at ***π***. For POM, $y^{*}=D_{\nu }\left(\tilde{y}-\hat{\nu }\right)+X\hat{\beta }$ , where ***D***_*ν*_ = *∂* ***η***/*∂* ***ν***. $W={\left(D_{\nu }V_{\nu }D_{\nu }\right)}^{-1}$ , where ***V***_*ν*_ is the variance-covariance matrix of the cumulative probability ***ν***.

For clustered study design, we write model (Eq. 6) in matrix notations

η=Xβ+Ub+h, (10)

where each component has three levels - category, cluster, and observation, except for ***β*** and ***b***. Please refer to Supplementary Section 1.2 of the online Supplementary Material for details of the model structure. Similarly, by applying pseudo-likelihood approach (Wolfinger and O’connell, 1993), the test statistic is

$$Q_{2}={\left(y^{*}-X\hat{\beta }\right)}^{\prime }\Sigma^{-1}K^{*}\Sigma^{-1}\left(y^{*}-X\hat{\beta }\right),$$ (11)

For GLMM, $y^{*}=D_{\pi }\left(y-\hat{\pi }\right)+X\hat{\beta }+U\hat{b}$ , $\hat{\pi }$ is a vector of fitted values returned by the null model ***η*** = ***Xβ*** + ***Ub***, and $\hat{\beta }$ is a vector of estimated coefficients of the fix effect, $\hat{b}$ is a vector of predicted values of ***b***. **Σ** = ***W*** ^−1^ + ***UG*** * ***U*** ′, where ***W*** ^−1^ = ***D***_*π*_ ***V***_*π*_ ***D***_*π*_, and ***G*** * is a (*J* − 1) × (*J* − 1) block matrix with entries ***I***_*n*_ ⊗ ***G***_*jh*_, *j*, *h* = 1, … , *J* − 1. For POMM, $y^{*}=D_{\nu }\left(\tilde{y}-\hat{\nu }\right)+X\hat{\beta }+U\hat{b}$ , ***W*** ^−1^ = ***D***_*ν*_ ***V***_*ν*_ ***D***_*ν*_ and ***G*** * is a (*J* − 1) block diagonal matrix with entries ***I***_*n*_ ⊗ ***G***_*jj*_.

### 2.4 *p*-Value Calculation

While deriving the test statistics for *Q*_1_ and *Q*_2_ is relatively straightforward in the pseudo-likelihood framework (as detailed in Supplementary Section 1 of the online Supplementary Material), obtaining their null distributions to calculate *p*-values is never an easy task. A major challenge lies in that classic asymptotic results in the likelihood framework tend to be inaccurate due to the relatively small sample size in microbiome studies (e.g., less than few hundred) and the over-dispersion in microbiome data (Chen et al., 2016). Small-sample correction procedures are available within relatively easier models such as the linear regression models or linear mixed model in literature (Chen et al., 2016; Zhan et al., 2017a; Zhan et al., 2018; Zhan et al., 2021). Yet, such an attempt in the more-complicated models (e.g., GLM, POM, GLMM, and POMM) considered in the current paper does not work out due to mathematical complexities of these models (e.g., canonical links are often unavailable or very complicated in such models). To this end, we resort to a pseudo-permutation strategy (Zhan et al., 2017b) to obtain accurate *p*-values in finite samples.

Briefly, the null distribution of all permutations of the test statistic can be approximated by the Pearson type III density, which is achieved by matching the first three moments. This strategy leads to a fast *p*-value calculation since we only need to use the matched Pearson type III density for *p*-value calculation without the need to draw real permutations (Zhan et al., 2017b). Essentially, we observe that the test statistics *Q*_1_ and *Q*_2_ can be reformulated as the trace of the product of two kernels matrix: a kernel matrix for outcomes (***K***_*Y*_) and a kernel matrix for microbiome data (***K*** in Eq. 7). Here we still assume that the kernel matrix for microbiome data is identical across multiple categories. Therefore, we use ***K*** instead of the original ***K*** * = ***I***_*J*−1_ ⊗ ***K*** in test statistics *Q*_1_ (Eq. 9) and *Q*_2_ (Eq. 11). In the proposed framework, let the weighted residual ***ϵ*** = ***W*** (***y*** * − ***Xβ***) for independent data or ***ϵ*** = **Σ** ^−1^ (***y*** * − ***Xβ***) for longitudinal data. The outcome kernel will be $K_{Y}=\tilde{\epsilon }{\tilde{\epsilon }}^{\prime }$ , where $\tilde{\epsilon }=\left(\epsilon_{1},\ldots ,\epsilon_{J-1}\right)$ is an *N* × (*J* − 1) matrix, where ***ϵ***_*j*_ is the weighted residuals for the *j*-th category. Originally, $\epsilon =Vec\left(\tilde{\epsilon }\right)$ is a vector of length *N* (*J* − 1), where *Vec* (⋅) denotes the operator that transforms a matrix into a column vector by vertically stacking the columns of the matrix. We refer the readers to previous publications for further details of *p*-values using the Pearson type III distribution (Zhan et al., 2017b).

Finally, recall that *p*-values of tests using different microbiome kernels could vary greatly depending on whether the kernel of choice captures the true underlying association pattern. To this end, we propose an omnibus test that first conducts individual tests using one of the kernels (Bray-Curtis, UniFrac, weighted UniFrac etc). And then combines these individual *p*-values (corresponding to different microbiome kernels) using the harmonic mean *p*-value (HMP) procedure (Wilson, 2019) for an omnibus *p*-value, based on which to conclude our inference of statistical association. This approach tends to be robust: it loses little power compared to when the best kernel (which is unknown in practice) is used and gains substantial power compared to when a poor choice of kernel is used.

## 3 Results

### 3.1 Simulation Studies

#### 3.1.1 Design of Simulations

We conducted comprehensive simulations to evaluate empirical type I error of MiRKAT-MC when there is no true associations, and statistical powers under different association patterns. For both independent and clustered study designs, microbiome compositions were simulated similarly as in previous studies (Zhao et al., 2015). Briefly, we first fitted a Dirchlet-multinomial distribution to a real upper-respiratory-tract microbiome dataset (Charlson et al., 2010), which contains 856 OTUs for 60 samples, and estimated the mean and dispersion parameters. We then used these estimated parameters to generate microbiome read counts via the Dirchlet-multinomial distribution. We intended to investigate what the most powerful kernel is when the causal OTUs are with or without phylogenetic relationships, and whether the abundance matters.

##### 3.1.1.1 Independent Data

We considered simulations when there are three categories (*J* = 3) and when there are five categories (*J* = 5). Data from each sample was simulated independently, according to following model

$$\eta_{ji}=\alpha_{j}+0.5\times x_{i1}+0.5\times x_{i2}+\beta \times scale\left(\underset{a\in A}{\sum }z_{ia}\right),$$ (12)

where *i* = 1, … , *N* and *j* = 1, … , *J* − 1. We set the sample size *N* = 80 or 200 for when *J* = 3, and *N* = 150 or 300 when *J* = 5. We simulated both nominal and ordinal outcomes, using appropriate link functions of *η*. For nominal data (GLM), *α*_*j*_ = −2, and for ordinal data (POM), *α*_*j*_ = *j* − 4. *x*_*i*1_ is a Bernoulli variable with probability of 0.5, whereas *x*_*i*2_ is a standard normal variable with mean 0 and variance 1. A is a set of outcome-associated OTUs among the *p* OTUs in the community. *β* = 0 for type I error simulations, for which the choice of A doesn’t matter. *scale* is the operation that standardize the data to be mean 0 and variance 1 across all the samples.

For statistical power evaluation, we considered three scenarios. Under the first two scenarios, causal OTUs (in A ) were selected from clusters of related taxa on a phylogenetic tree. In specific, we first partitioned the simulated OTUs into 20 clusters through the partitioning-around-medoids (PAM) algorithm based on the corresponding phylogenetic tree. For scenario 1, we randomly chose a common cluster of the OTUs as the causal OTUs. For scenario 2, we chose the rarest cluster as the causal OTUs. For scenario 3, we picked the 10 most abundant OTUs without consideration of phylogenetic information. These three scenarios correspond to situations in which the weighted UniFrac, unweighted UniFrac and the Bray-Curtis distances are expected to be the most powerful, respectively. For scenarios 1 and 3, *β* = 0.6, 0.8, 1.2, 1.6, 2.0, and *β* = 2, 4, 6, 8, 10 for scenario 2.

For each scenario, we employed the weighted UniFrac (*K*_*w*_), the unweighted UniFrac (*K*_*u*_), the Bray-Curtis (*K*_*BC*_) and a generalized UniFrac kernel with the parameter of 0.5 (*K*_5_) for association testing. We also conducted the omnibus test by combining the *p*-values from all individual tests. To obtain convincing results, we generated 10,000 replicates to estimate the empirical type I errors and 2,000 replicates for statistical powers. Statistical significance was established under the nominal level of *α* = 0.05 for all the simulation studies.

##### 3.1.1.2 Clustered Data

We simulated two scenarios to assess MiRKAT-MC when data is clustered. We simulated a family based study and a longitudinal study. For family-based data, we included only a random intercept in the model to capture the correlation between samples, while for longitudinal data, both a random intercept and a random slope of time were involved in the model. We set the number of clusters *n* = 30 or 60 for three categories (*J* = 3), and *n* = 50 or 100 for five categories (*J* = 5). We simulated data under an unbalanced design: i.e., clusters may have a different number of observations. To achieve this, *n*/2 of the clusters have three observations and the other *n*/2 of the clusters have four observations. In this way, the total numbers of observations are *N* = 105 (*n* = 30) and *N* = 210 (*n* = 60) when *J* = 3 and *N* = 175 (*n* = 50) and *N* = 350 (*n* = 100) when *J* = 5. Within each cluster, the outcome category may vary over observations; e.g., in longitudinal studies, a person may be of one disease category at one time point and of a different disease category at a different time point.

The following model was utilized to simulate the data

$$\eta_{jik}=\alpha_{j}+0.5\times x_{ik1}+0.5\times x_{ik2}+u_{ik}^{\prime }b_{ji}+\beta \times scale\left(\underset{a\in A}{\sum }z_{ika}\right),$$ (13)

where *i* = 1, … , *n*, *j* = 1, … , *J* − 1, and *k* = 1, … , *m*_*i*_. The definition of the parameters *η*, *α*_*j*_, *β x*_*ik*1_, *x*_*ik*2_, A and *scale* function are identical to the counterparts in model (Eq. 12). The same three scenarios of choices of A were considered for power assessment. When the model included only a random intercept, *u*_*ik*_ = 1 and *b*_*ji*_ was generated from $∼N\left(0,g_{jj}\right)$ , where $g_{jj}=\frac{1}{4},1,4$ being the variance, respectively. When considering both a random intercept and a random slope of time, $u_{ik}={\left(1,t_{ik}\right)}^{\prime }$ and *b*_*ji*_ was simulated from $N\left(0,G_{jj}\right)$ , where $G_{jj}=\left(\begin{array}{cc} g_{jj11} & g_{jj12}\\ g_{jj21} & g_{jj22} \end{array}\right)$ . We set $g_{jj11}=g_{jj22}=\frac{1}{4},1,4$ , respectively, and *g*_*jj*12_ = *g*_*gg*21_ were determined by $\frac{1}{2}g_{jj11}$ . Thus, the correlation between the random intercept and the random slope was fixed at $\frac{1}{2}$ . The generation of random effect ***b***_*ji*_ was different for GLMM and POMM. Specifically, for a fixed cluster *i*, for GLMM, we generated a new random vector of ***b***_*ji*_ for each category *j* from the above distribution. For the ease of data generation, we kept ***G***_*jj*_ the same across categories and did not consider correlation of ***b***_*ji*_ between categories for nominal data. However, as we discussed in model (Eq. 3), GLMM enjoys the freedom of different ***G***_*jj*_ and correlated ***b***_*ji*_ across different categories. In contrast, for POMM, we generated a new random vector of ***b***_*i*_ only once for each cluster *i* and then plugged the same ***b***_*i*_ in model (Eq. 13) for different categories.

#### 3.1.2 Simulation Results

Empirical type I error rates of MiRKAT-MCN (for nominal outcomes) and MiRKAT-MCO (for ordinal outcomes) for independent data are reported in Table 1. As seen in the table, the empirical type I errors (at *α* = 0.05) of MiRKAT-MC are all very close to the expected level. Empirical type I error rates under different mixed models for clustered data are reported in Supplementary Tables S1–S4 (Supplementary Section 2.1, online Supplementary Material), which also show well-controlled type I errors for both nominal and ordinal outcomes.

**TABLE 1:** Empirical type I error rates of MiRKAT-MC for independent data with three-categories.

	MiRKAT-MCN		MiRKAT-MCO	
	*N* = 80	*N* = 200	*N* = 80	*N* = 200
K_w_	0.0463	0.0465	0.0440	0.0470
K_u_	0.0436	0.0491	0.0487	0.0492
K_BC_	0.0488	0.0468	0.0469	0.0449
K_5_	0.0479	0.0518	0.0476	0.0466
HMP	0.0502	0.0475	0.0461	0.0455

*N* denotes the sample size. K_w_, the weighted UniFrac kernel; K_u_, the unweighted UniFrac kernel; K_BC_, the Bray-Curtis kernel; K_5_, the generalized UniFrac kernel with parameter 0.5; HMP, the omnibus test using harmonic mean *p*-value test.

**FIGURE 1:**
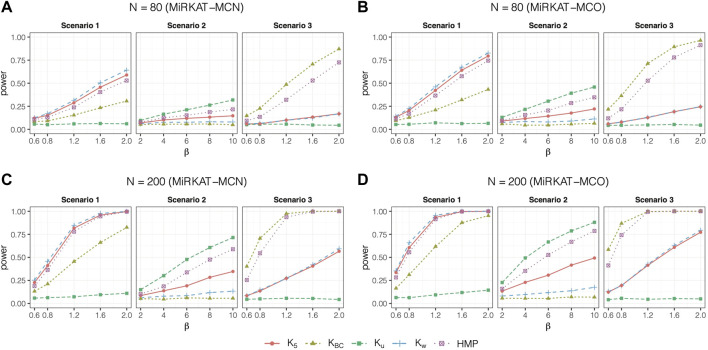
Statistical powers of MiRKAT-MC for independent data with three categories. Scenario 1: A = A randomly selected common cluster among 20 clusters by PAM; Scenario 2: A = The rarest cluster among 20 clusters by PAM; Scenario 3: A = 10 most abundant OTUs. K_w_, the weighted UniFrac kernel; K_u_, the unweighted UniFrac kernel; K_BC_, the Bray-Curtis kernel; K_5_, the generalized UniFrac kernel with parameter 0.5; HMP, the omnibus test using harmonic mean *p*-value test. **(A)** MiRKAT-MCN with 80 total samples; **(B)** MiRKAT-MCO with 80 total samples; **(C)** MiRKAT-MCN with 200 total samples; **(D)** MiRKAT-MCO with 200 total samples.

Figure 1 shows the statistical powers of MiRKAT-MC using independent data with three categories. The results with five categories using independent data are in Supplementary Figure S1 (Supplementary Section 2.2, online Supplementary Material). We observe that the tests with weighted UniFrac, unweighted UniFrac, and Bray-Curtis kernels are most powerful for scenarios 1, 2, and 3, respectively, regardless of whether the outcome is nominal or ordinal. However, the tests with Bray-Curtis kernel produced very little power in scenario 2, and the tests with unweighted UniFrac showed little power in scenario 3: the statistical power are close to their expected type I error. This is due to the differences in the true association signals that each of the kernels is designed to capture. The weighted UniFrac kernel is most powerful to capture signals that are dominated by common taxa in a cluster on a phylogenetic tree, while the unweighted UniFrac kernel shows its strengths when rare OTUs in a phylogenetic cluster determine the association (Chen et al., 2012). In contrast, the Bray-Curtis kernel is more appropriate when the outcome is associated with a set of OTUs with high abundance without referring to a phylogenetic tree. The Omnibus test considering all four kernels is robust. For example, among the tests using single kernels, only Bray-Curtis kernel shows significant powers under scenario 3. Yet, the omnibus test is still able to detect the association.

Table 2 shows the empirical type I error for our proposed methods when the data are clustered. Again, type I errors are well controlled to their nominal level. The statistical powers for simulations when data is clustered are presented in Supplementary Figures S2–S5 (Supplementary Section 2.2, online Supplementary Material). Under three categories, Supplementary Figure S2 corresponds to models with random intercepts, while Supplementary Figure S3 presents models with both random intercepts and random slopes. Similarly, Supplementary Figure S4 corresponds to models with random intercepts with five categories; Supplementary Figure S5 is about models with both random intercepts and random slopes with five categories. The conclusions are similar to those of independent data. In addition, we observe that given a simulation scenario, a choice of kernel and an effect size, when the variance of the random effect (elements in ***G***_*jj*_ in Eq. 13) increases, the statistical power decreases. It is because with the increase of the random effects, the within-cluster correlation increases, leading to a lower effective sample size.

**TABLE 2:** Empirical type I errors of MiRKAT-MC for clustered data with a random intercept and a random slope model with three-category outcomes.

	*n* = 30 (*N* = 105)			*n* = 60 (*N* = 210)		
*g*	0.25	1	4	0.25	1	4
MiRKAT-MCN						
K_w_	0.0498	0.0492	0.0467	0.0478	0.0496	0.0484
K_u_	0.0521	0.0533	0.0486	0.0449	0.0508	0.0478
K_BC_	0.0519	0.0542	0.0494	0.0522	0.0478	0.0497
K_5_	0.0527	0.0516	0.0521	0.0521	0.0468	0.0505
HMP	0.0514	0.0533	0.0472	0.0465	0.0478	0.0488
MiRKAT-MCO						
K_w_	0.0500	0.0473	0.0474	0.0449	0.0498	0.0457
K_u_	0.0486	0.0506	0.0487	0.0483	0.0483	0.0538
K_BC_	0.0535	0.0507	0.0487	0.0453	0.0493	0.0485
K_5_	0.0519	0.0471	0.0489	0.0476	0.0501	0.0486
HMP	0.0495	0.0467	0.0481	0.0452	0.0483	0.0475

*n* indicates the number of clusters while *N* is the number of total observations. *g* denotes the variance of random effects. The definition of K_w_, K_u_, K_BC_, K_5_, and HMP is the same as Table 1.

### 3.2 Real Data Analysis

#### 3.2.1 Associations Between Antibiotic Exposure and Gut Microbiome in Non-Obese Diabetic Mice in a Longitudinal Study

In the original study (Livanos et al., 2016), 555 non-obese diabetic mice were randomly assigned to three groups with each group exposed to distinct patterns and doses of antibiotics. The mice that were born to the same female and that were of the same sex constituted a cluster and each cluster received the same treatment. The first group (51 clusters, 203 mice) received sub-therapeutic continuous (STAT) antibiotic exposure, the second group (42 clusters, 167 mice) received therapeutic-dose pulsed (PAT) antibiotic exposure, and the last group (47 clusters, 135 mice) was not exposed to antibiotics and served as the control group (Hu et al., 2020). Microbiome data from fecal, cecal or ileal samples were collected longitudinally for each cluster by sacrificing a mouse, at 3, 6, 10, and 13 weeks from the start of the experiment (week 0). The number of observations per cluster varied from 2 (i.e., at week 3 and 6) to 4 (i.e., at week 3, 6, 10, and 13).

The goal of this application is to test the association between treatment groups (STAT, PAT or control) and gut microbiome. Here, we exclusively analyzed the fecal samples, leaving 499 samples from 140 clusters over time. The gut microbiome was profiled from each sample and the raw sequence data is available on the Qiita database (study ID 10508). Specifically, the V4 region of the bacterial 16S rRNA gene was PCR amplified, followed by performing paired-end sequencing of the amplicon library. We reprocessed the pre-joined and trimmed sequencing data through DADA2 pipeline in R (Callahan et al., 2016). As a result, the amplicon sequence variant (ASV) table was constructed. After removing chimeras identified by consensus across samples, the table contained 3031 ASVs. The ASV table was rarefied to an equal depth of 5,000 for each sample. We then assigned taxonomy based on Ribosomal Database Project’s (RDP) training set 16, and constructed a phylogenetic tree using R package “phangorn” (Schliep, 2010). The tree was rooted by specifying the middle tip (i.e., 1515) as the outgroup. We calculated the UniFrac distance based on the rooted tree and the rarefied ASV table with the “GUniFrac” R package (Chen et al., 2012).

**FIGURE 2:**
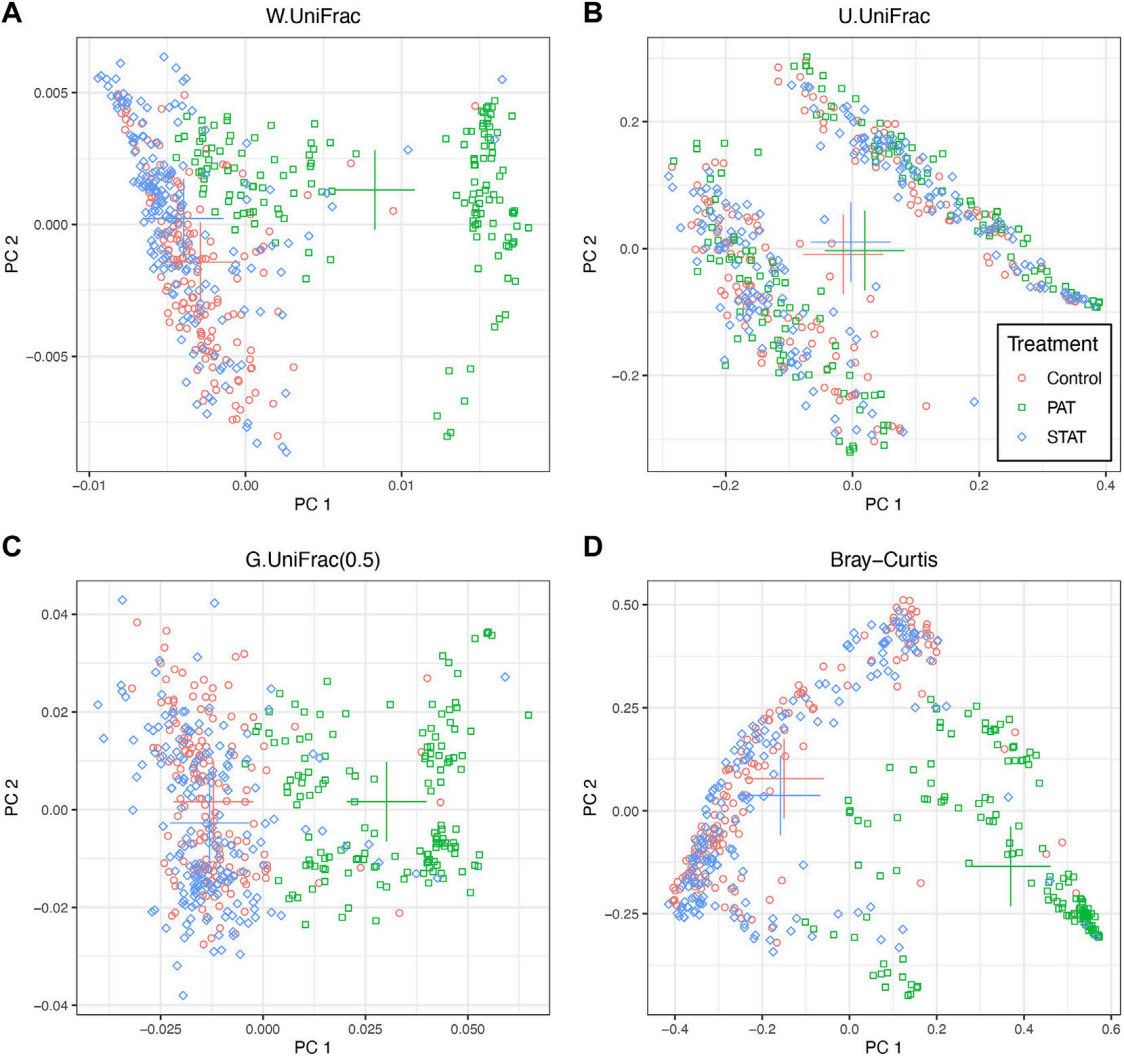
The two-dimensional PCoA plots depicting microbiome composition for different antibiotic treatment groups under various dissimilarity measures. All 499 fecal samples are included in the plots. PAT, therapeutic-dose pulsed antibiotic exposure; STAT, sub-therapeutic continuous antibiotic exposure. The crosses denote the centroid of points of each treatment group. **(A)** W.UniFrac: weighted UniFrac distance; **(B)** U.UniFrac: unweighted UniFrac distance; **(C)** G.UniFrac(0.5): generalized UniFrac distance with tuning parameter *a* = 0.5; **(D)** Bray-Curtis: Bray-Curtis dissimilarity.

Here we first visually checked the relationship between gut microbiome composition and antibiotic treatment groups under different dissimilarity measures with PCoA plots (Figure 2). All 499 fecal samples are included in the plot, although they might be collected at different time points. Microbiome composition of the PAT group is clearly separated from that of the STAT group and that of the control group, under weighted UniFrac distance, generalized UniFrac distance and Bray-Curtis dissimilarity. However, under unweighted UniFrac distance, it is hard to distinguish the microbiome compositions of three treatment groups since they are clustered at two areas.

To show the performance of MiRKAT-MCN on independent nominal data, we selected samples at week 3 only. All 140 clusters had microbiome data available. By setting treatment groups as the dependent variable and adjusting for gender of mice, we observed very significant association between gut microbiome and the antibiotic treatment groups using weighted, unweighted, and generalized UniFrac kernels, Bray-Curtis kernel, and the omnibus test (all *p*-values <0.0001 ). To better show the performance of the proposed model, and since the sample sizes of microbiome studies are usually smaller, we randomly subsampled 90 samples from the 140 samples at week 3. The down-sampled data consisted of 41 male and 49 female mice, and there were 36, 22, and 32 mice in the STAT, PAT and control groups, respectively. With the reduced sample size, all tests, including the tests using each of the kernels and the omnibus test, identified significant association between microbiome and antibiotic treament, with all *p*-values less than 0.0001, except for when using the unweighted kernel (*p*-value = 0.01).

We also applied MiRKAT-MCN for clustered data to this study. Similarly, we randomly selected 30 clusters with 105 samples (17 male and 13 female mice clusters) from the original dataset for analysis, where there were 15, 6, and 9 clusters in STAT, PAT, and control group, respectively. We applied MiRKAT-MCN for clustered data to evaluate the association between antibiotic treatment and microbiome, adjusting for sex and time (in weeks), and accounting for the cluster-specific correlation through a random intercept and a random slope of time. Again, we employed the same kernels as above and the omnibus test for analysis. Apart from the test using the unweighted UniFrac kernel with *p*-value only 0.03, all other tests were highly significant with *p*-values less than 0.001.

These two analyses indicate that antibiotic exposure during early life did alter the microbiome composition in non-obese diabetic mice, no matter we stared at the week 3 or inspected over time. Moreover, the disparities of *p*-values by using different kernels, although all significant, suggest that the antibiotic use may have affected the relative abundance of OTUs, because the unweighted UniFrac kernel, which only accounts presence/absence of taxa and gives higher weight to rare taxa, provides the least significant result.

#### 3.2.2 Associations Between Obesity and Gut Microbiome in a Family-Based Study

A study was conducted by Goodrich et al. (Goodrich et al., 2014) to investigate the role of host genetics on gut microbiome, and their impact on host phenotype, such as the body mass index (BMI). Fecal samples were collected from families in the United Kingdom. The V4 region of 16S rRNA gene was sequenced to identify the microbiome composition. The raw data was downloaded from the European Bioinformatics Institute (EBI) with accession numbers ERP006339 and ERP006342. We used QIIME (version 1.9.0-dev) (Caporaso et al., 2010) to assign the sequencing tags to 7,365 non-singleton OTUs at 97*%* similarity using the reference-based OTU-picking approach, and to generate a rooted phylogenetic tree. All samples were rarefied to 10,000 counts per sample before calculating the distance measures.

For this analysis, we focused on 311 samples from 145 monozygotic twin pairs. All the twins were female, aged from 27 to 83 with an median age of 63. In order to compare the performance of different methods, we treated the BMI as continuous, binary, three-category ordinal and three-category nominal data, and applied CSKAT (Zhan et al., 2018), GLMM-MiRKAT (Koh et al., 2019), MiRKAT-MCO and MiRKAT-MCN for each outcome type, respectively. CSKAT was developed for microbiome association analysis of clustered/longitudinal study for continuous outcomes while GLMM-MiRKAT was for the similar association analysis for binary and count outcomes, respectively. For binary outcome, we classified the study participants into a non-obese (248 samples) and an obese group (63 samples) based on BMI < 30 or BMI ≥30. For the three-category outcome, we classify study participants into normal (BMI < 25), overweight (25 ≤ BMI < 30), and obese (BMI ≥30) groups, where there were 147, 101, and 63 samples in each group, respectively. We can treat the three categories as nominal or ordinal when applying MiRKAT-MC. For all the analyses, we assessed the microbiome-BMI (or BMI category) association, adjusting for age and including a twin-level random intercept to capture the within-twin-pair correlations due to common genetic, biological and other environmental factors. The weighted, unweighted, generalized UniFrac distance and the Bray-Curtis distance were used to construct kernel functions based on Eq. 7. The test statistics of CSKAT and GLMM-MiRKAT followed the original papers, but we used the same technique as MiRKAT-MC to calculate *p*-values, in order to ensure comparability.

**FIGURE 3:**
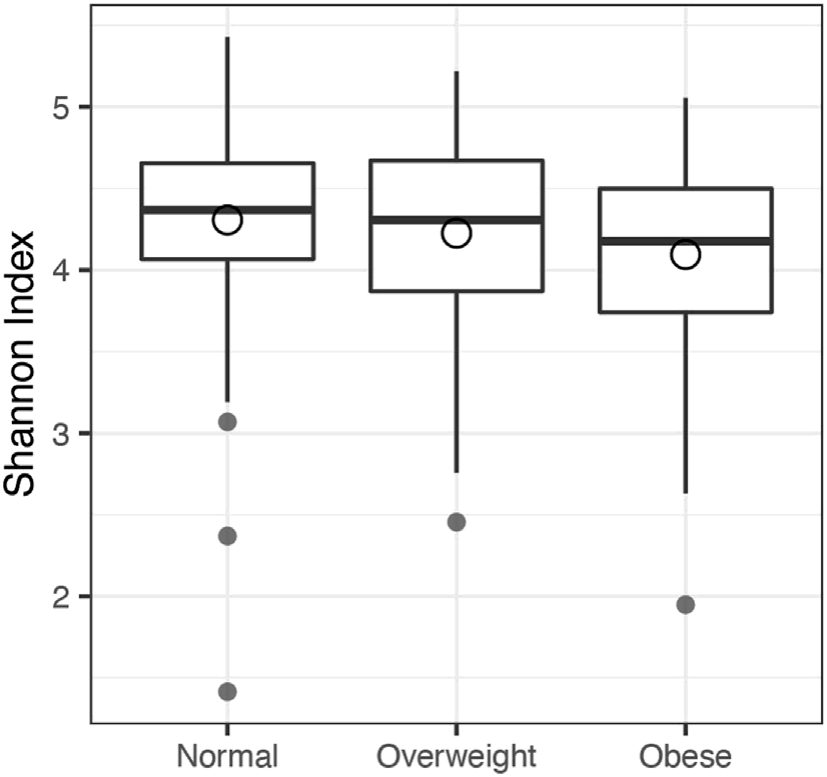
The boxplot of Shannon index across BMI categories in United Kingdom twins study. Normal: BMI < 25; Overweight: 25 ≤ BMI < 30; Obese: BMI ≥30. The circle on each box denotes the mean of Shannon Index in that category.

Figure 3 compares the microbiome Shannon index across the three BMI categories. The decreasing trend of Shannon index from the normal category to the obese category implies that higher BMI may reduce the microbiome diversity. The results of association analyses are shown in Table 3, where the smallest significant *p*-value of each kernel across four methods is bolded. At the first glance, all the individual tests provided significant association at type I error of 0.05 except when the weighted UniFrac kernel was used. The omnibus test also provided significant association. However, MiRKAT-MCO gave the smallest *p*-values when using the unweighted UniFrac, the generalized UniFrac and the omnibus test. MiRKAT-MCO was always more powerful than MiRKAT-MCN in this analysis, which is reasonable because MiRKAT-MCO utilized the order information in data. Both MiRKAT-MCO and MiRKAT-MCN were more powerful than GLMM-MiRKAT except when the weighted UniFrac kernel was used, for which none of the methods was significant. Our results are also consistent with the conclusion of the previous study (Zhan et al., 2018) that the unweighted UniFrac kernel and the Bray-Curtis kernel were most suitable for this dataset.

**TABLE 3:** *p*-values of testing for the BMI-microbiome association in United Kingdom twins dataset using different methods and kernels.

	CSKAT	GLMM-MiRKAT-Binary	MiRKAT-MCO	MiRKAT-MCN
K_w_	0.1455	0.1750	0.2223	0.3268
K_u_	0.0036	0.0182	**0.0014**	0.0033
K_BC_	**0.0012**	0.0021	0.0016	0.0015
K_5_	0.0278	0.0370	**0.0194**	0.0264
HMP	0.0036	0.0075	**0.0030**	0.0040

The bold value is the smallest significant *p*-value across four methods given the kernel/method. The definition of K_w_, K_u_, K_BC_, K_5_, and HMP is the same as Table 1.

## 4 Discussion

Multi-categorical outcomes, both nominal and ordinal, are increasingly common in biological and biomedical research over recent years. Investigating the subtle microbiome composition differences among multiple subtypes of a disease provides a broad view of microbiome variation. It is typically a first step to a further study of microbiome functionality and other related topics. Additionally, clustered designs, as a supplement to population-based studies, have become very popular recently when researchers are interested in dynamic variations or the variations among related individuals. While the toolbox for analyzing data collected from population-based studies is plentiful, methods for analyzing these clustered data are underdeveloped. To fill these research gaps, we proposed MiRKAT-MC for testing for association between multi-categorical outcomes and microbial community compositions for both population-based and clustered/longitudinal studies.

Our major contributions in this paper are two-fold. First, we have successfully used the generalized logit model and the proportional odds model to enable direct association analysis between multi-categorical outcomes and microbiome compositions, without the need of combining categories or conducting pairwise comparisons. Existing approaches either compare two categories at a time and then conduct multiple testing correction, or combine multiple groups into a single category and compare it to the baseline. The pair-wise comparison approach tends to lose power due to the burden of multiple comparison. In addition, combining multiple groups into a single category can lead to substantial power loss when the microbiome effects on the categories are in opposite directions. However, when we have more than two categories, MiRKAT-MC can incorporate the heterogeneity in microbiome data and compare all non-reference categories to the reference category. Comparing to the potential alternative approach that first compares each pair of categories followed by multiple comparison adjustment, MiRKAT-MC would be much more powerful. Moreover, the new association analysis framework in the proportional odds model is extremely appealing for ordinal outcome data, as none of the existing approaches takes advantage of the order information in this particular type of data. Second, we have adapted a fast pseudo-permutation strategy previously developed under linear models to more complicated GLM(M) and POM(M) to achieve efficient and accurate *p*-values calculation. Unlike the ascendants which calculate *p*-values through either asymptotic distribution or direct permutation among exchangeable clusters, MiRKAT-MC controls type I error perfectly, even when the sample size is small, yet avoids the time-consuming and complex permutation.

As a non-parametric distance-based method, MiRKAT-MC comes with some limitations. First of all, the choice of distance metrics is subjective and could impact its performance. To this point, we propose to conduct analysis using multiple kernels/distances, generate multiple *p*-values and combine them via the harmonic mean approach (Wilson, 2019). Secondly, like other community level analysis of microbiome (Anderson, 2001; Zhao et al., 2015; Tang et al., 2016; Koh et al., 2019), MiRKAT-MC aggregates information across all taxa to form a community level test. This usually serves as the first step in understanding microbiome-phenotype relationship. However, these approaches do not provide insight on which taxa are driving the overall association. Thirdly, we used microbiome beta-diversity to define our distance/kernel matrix, which is convenient and proven useful. Many beta-diversities have been proposed and widely used in microbiome studies, which capture distinct characteristics of the underlying association pattern (see (Plantinga et al., 2017)). However, recent literature indicated that the structure of microbiome community may vary even when their diversities and compositions are comparable. In that context, if we are able to develop a sample-to-sample distance matrix that captures the important structure variations, such distance can be easily incorporated into our framework. Developing a kernel/distance for subtle structural differences in microbiome communities can be an interesting scientific endeavor, however, it is beyond the scope of this paper.

Computational efficiency of MiRKAT-MC is investigated and reported in Table 4. MiRKAT-MC is extremely fast when dealing with independent data. When data is clustered, the computational time increases substantially, mainly because of the increased time in fitting the null GLMM/POMM in the presence of random effects. Nevertheless, the computational time for MiRKAT-MC is very manageable even with clustered data. Given that most microbiome studies are relatively small in sample size, for three-category data, MiRKAT-MC can usually be accomplished in 0.1 s for population-based studies with sample size less than 200, and in 7 s for clustered studies with total sample size less than 210.

**TABLE 4:** Computation efficiency of MiRKAT-MC. Each result is the average time of one association test averaged from running 100 replicate association tests.

		MiRKAT-MCN (s)	MiRKAT-MCO (s)
Independent data			
*J* = 3	*N* = 80	0.0150	0.0139
	*N* = 200	0.0914	0.0796
*J* = 5	*N* = 150	0.0978	0.0426
	*N* = 300	0.7627	0.2568
Longitudinal data			
*J* = 3	*n* = 30 (*N* = 105)	6.438	2.844
	*n* = 60 (*N* = 210)	6.672	2.994
*J* = 5	*n* = 50 (*N* = 175)	11.964	4.758
	*n* = 100 (*N* = 350)	26.328	15.252

For longitudinal data, both random intercepts and random slopes of time are included in the null models. The weighted UniFrac kernel was applied without loss of generalization. *n* denotes the number of clusters, whereas *N* is the total sample size. All the computation was conducted on a Macbook Pro (15-inch, 2019) laptop with 2.3 GHz 8-Core Intel Core i9 processor and 16 GB memory, without using parallel or other speed-up strategies.

In summary, we propose MiRKAT-MC, a microbiome regression association test for multi-categorical outcomes with independent and clustered study designs. The proposed methods show well controlled type I errors and high power over multiple scenarios through extensive simulations and better performance than competitors in real data analyses. It is easy to use and fast to compute. We believe that MiRKAT-MC will enrich the toolbox of researchers to conduct microbiome research with multi-categorical outcomes.

## Data Availability

The original contributions presented in the study are included in the article/Supplementary Material, further inquiries can be directed to the corresponding authors.
