# Supplementary figures and images for: MiRKAT-MC: A Distance-Based Microbiome Kernel Association Test With Multi-Categorical Outcomes
Source: Front Genet. 2022 Apr 1;13:841764. doi: 10.3389/fgene.2022.841764 (PMC9010828; doi:10.3389/fgene.2022.841764)

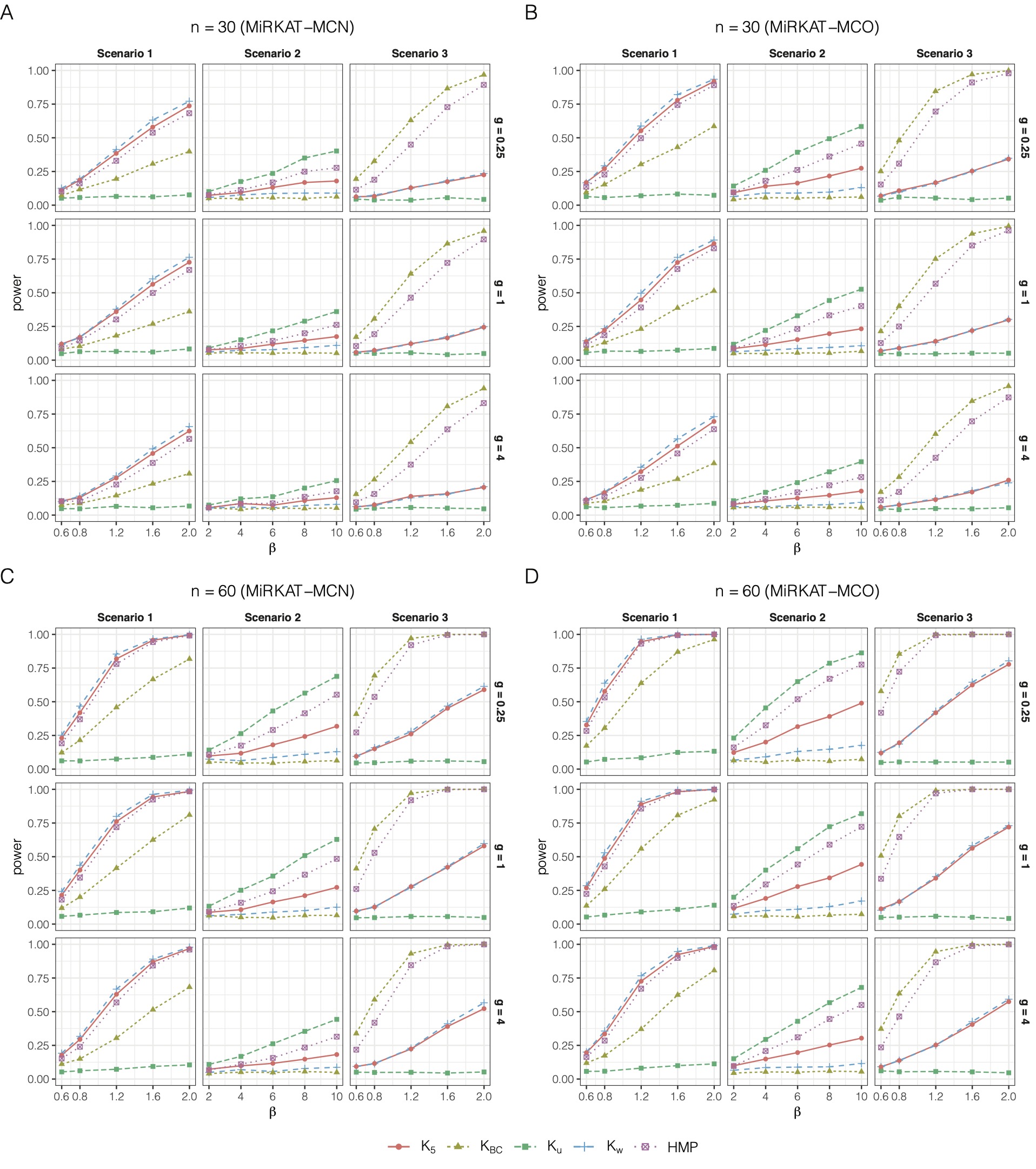

Supplement: Supplementary file 1 [file Image3.JPEG]

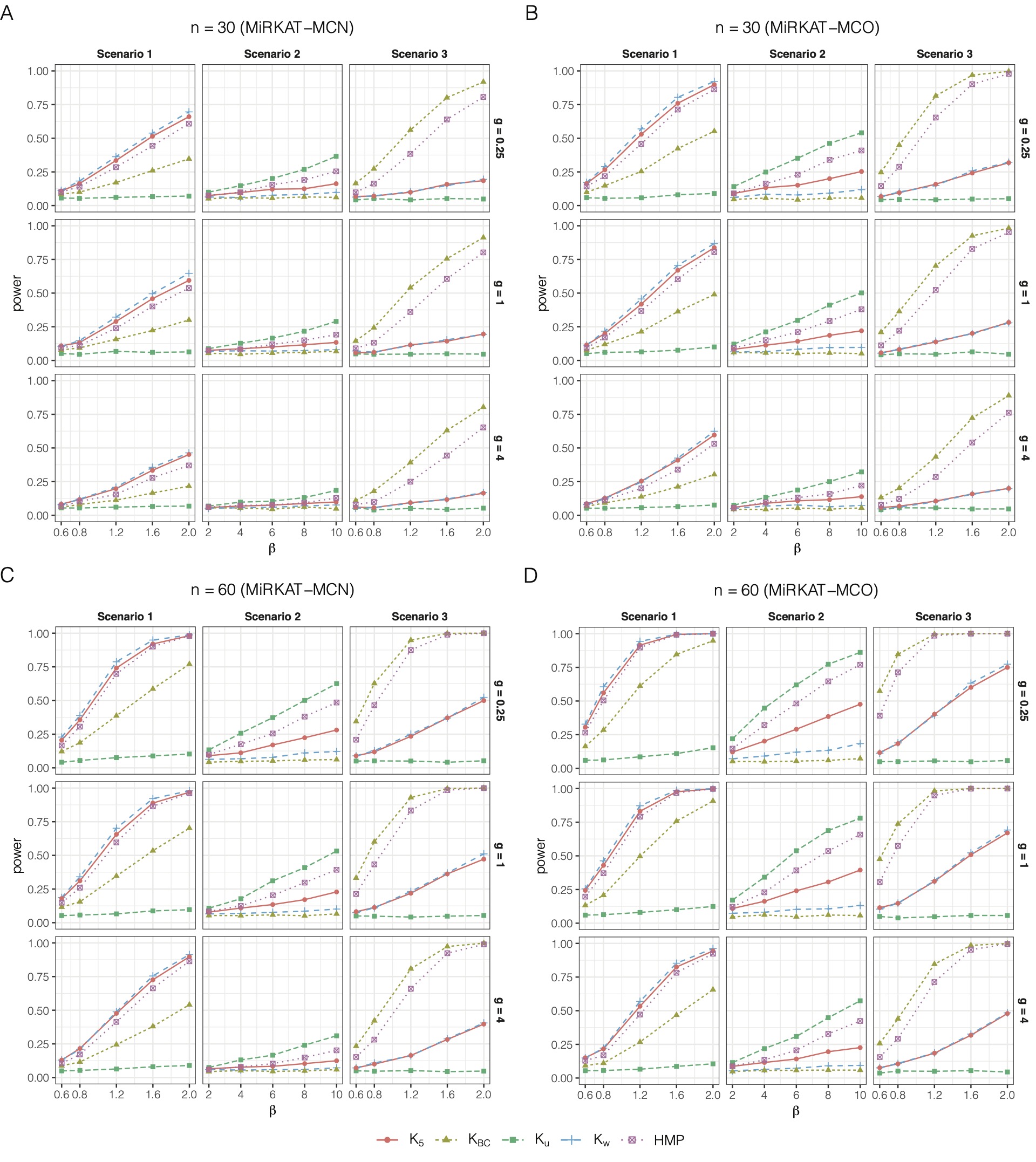

Supplement: Supplementary file 2 [file Image1.JPEG]

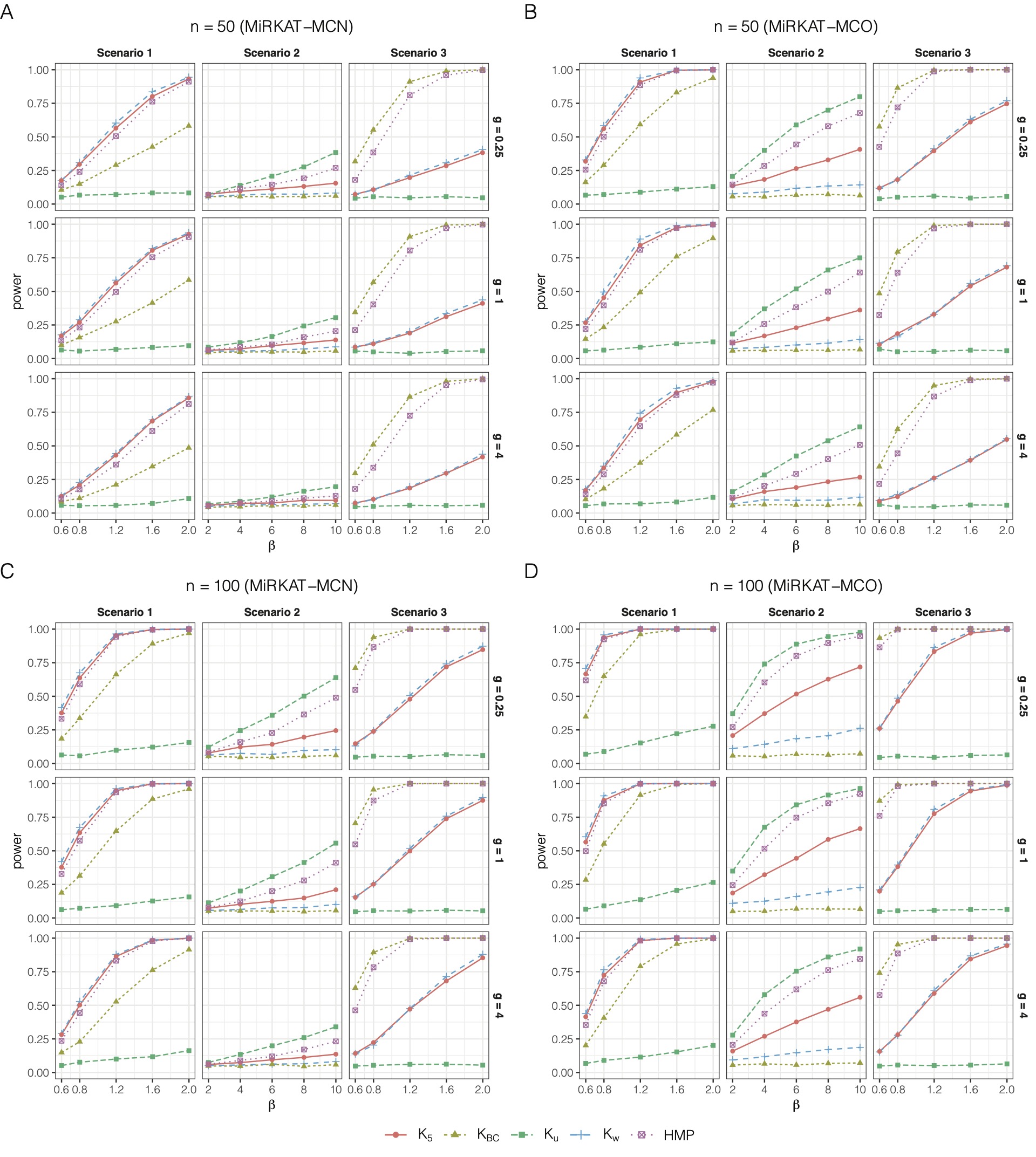

Supplement: Supplementary file 3 [file Image4.JPEG]

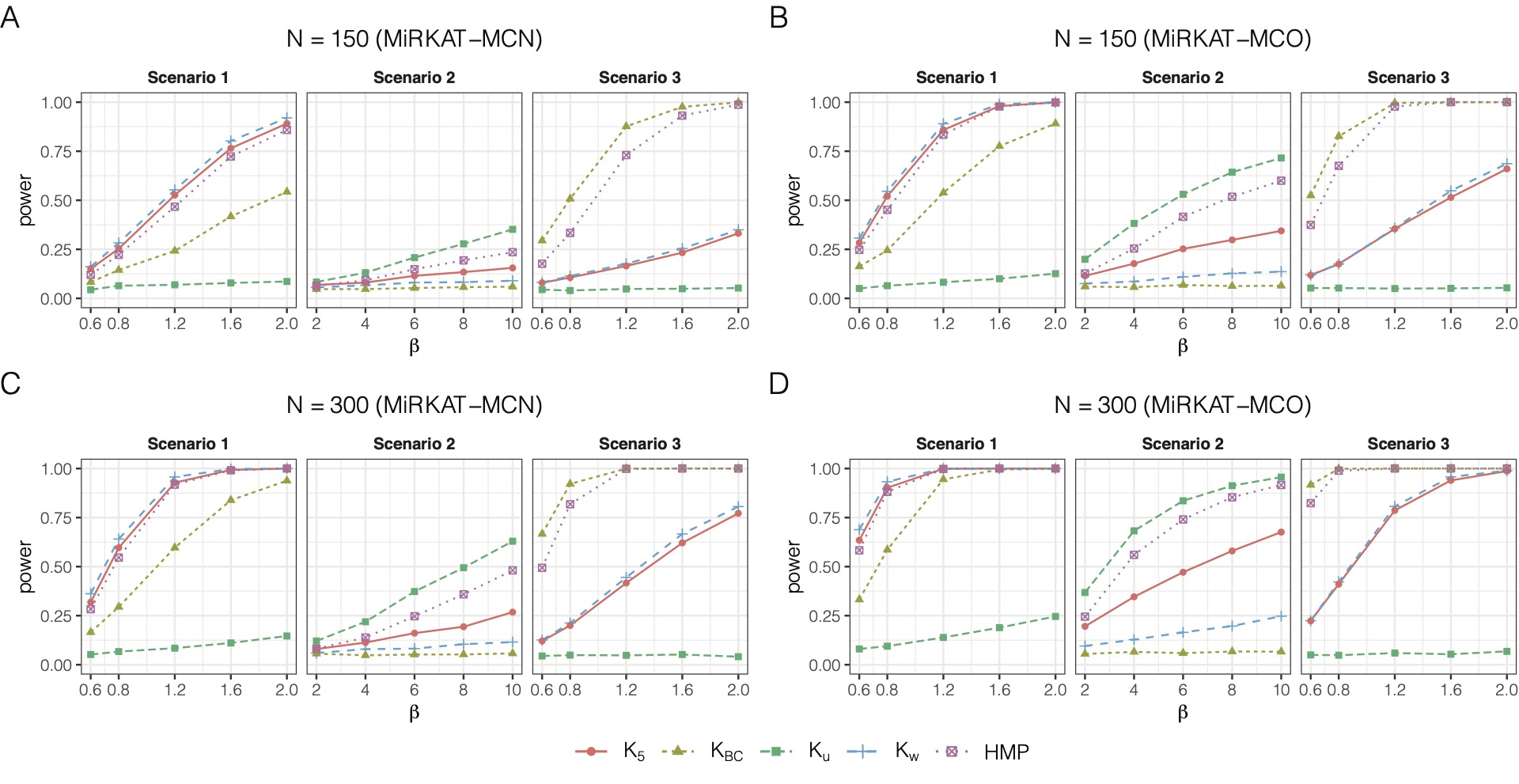

Supplement: Supplementary file 4 [file Image2.JPEG]

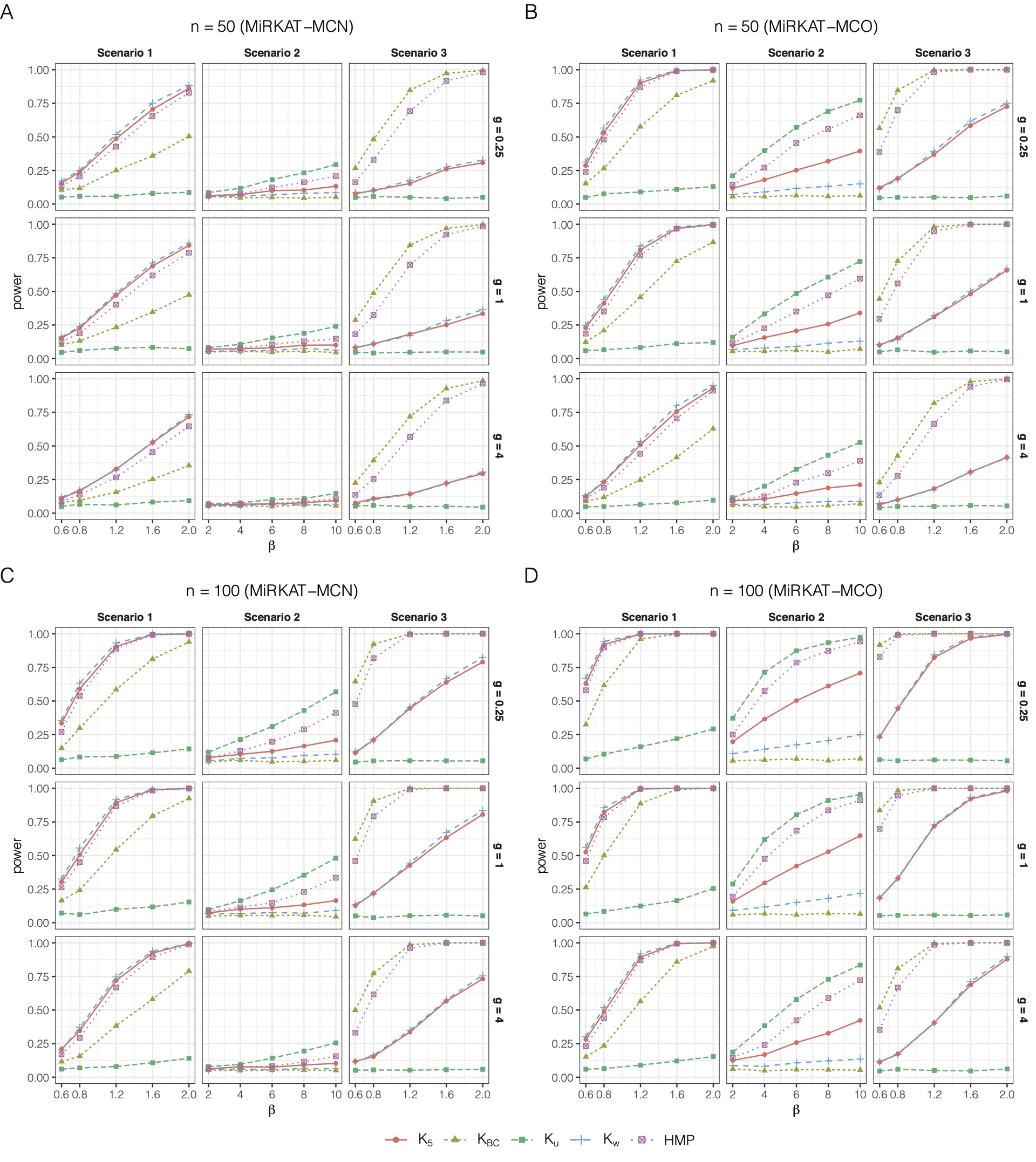

Supplement: Supplementary file 5 [file Image5.JPEG]
