# Supplementary material for: MiRKAT-MC: A Distance-Based Microbiome Kernel Association Test With Multi-Categorical Outcomes
Source: Front Genet. 2022 Apr 1;13:841764. doi: 10.3389/fgene.2022.841764 (PMC9010828; doi:10.3389/fgene.2022.841764)
Supplement: Supplementary file 6 [file DataSheet1.PDF]

Supplementary Material

1 DERIVATION OF MIRKAT-MC ASSOCIATION ANALYSIS

1.1 MiRKAT-MC for independent data

We developed the score test statistic for MiRKAT-MC using the pseudo-likelihood framework. We take MiRKAT-MCN as an example for demonstration, as MiRKAT-MCO can be developed in a similar way by using the corresponding link function and the cumulative probability. We will address some details of MiRKAT-MCO at the end of this section. Suppose we have N samples and each of them falls to one of the J categories. Let y_{ji}, a binary variable denotes whether the i-th sample belongs to the j-th category or not. That is, $y_{ji} = 1$ means sample i is of category j and otherwise, $y_{ji} = 0$. We consider the following models

$$\eta_{ji} = \log \frac{\pi_{ji}}{1 - \sum_{j=1}^{J-1} \pi_{ji}} = \alpha_j + \boldsymbol{x}_i' \boldsymbol{\beta}_j + h_{ji}, \tag{S1}$$

for $i = 1, \ldots, N$, $j = 1, \ldots, J-1$ and

$$y_{ji} = \pi_{ji} + e_{ji}, \tag{S2}$$

where $\pi_{ji} = E(y_{ji}|h_{ji})$; α_j is the intercept for category j; $\boldsymbol{x}_i = (x_{i1}, \ldots, x_{iq})'$ is a $q \times 1$ vector of covariates for sample i; $\boldsymbol{\beta}_j = (\beta_{j1}, \ldots, \beta_{jq})'$ is the corresponding $q \times 1$ vector of regression coefficients for category j. h_{ji} represents the effect of microbiome of sample i on the category j, with the distribution $\mathcal{N}(0, \tau K_i)$, where both $\tau, K_i \geq 0$. e_{ji} is the error term that $E(e_{ji}|\pi_{ji}) = 0$, $Var(e_{ji}|\pi_{ji}) = \pi_{ji}(1 - \pi_{ji})$ and $Cov(e_{ji}, e_{hi}|\pi_{ji}, \pi_{hi}) = -\pi_{ji}\pi_{hi}$ for $j \neq h$. The inverse link function $f(\cdot)$ of the generalized logit mixed model can be easily calculated as

$$\pi_{ji} = f(\eta_{ji}) = \frac{\exp(\eta_{ji})}{1 + \sum_{j=1}^{J-1} \exp(\eta_{ji})}. \tag{S3}$$

It is approximately linearized by the first-order Taylor series expension around a particular value $\bar{\eta}_{ji}$:

$$f(\eta_{ji}) = f(\bar{\eta}_{ji}) + \frac{\partial f(\bar{\eta}_{ji})}{\partial \bar{\eta}_{ji}} (\eta_{ji} - \bar{\eta}_{ji}). \tag{S4}$$

After doing some simple algebra, we have

$$y_{ji} - f(\bar{\eta}_{ji}) = \bar{d}_{ji}^{-1} (\eta_{ji} - \bar{\eta}_{ji}) + e_{ji}, \tag{S5}$$

where $\bar{d}_{ji}^{-1} = \frac{\partial f(\bar{\eta}_{ji})}{\partial \bar{\eta}_{ji}} = \frac{\bar{\pi}_{ji}\bar{\pi}_{Ji}}{\bar{\pi}_{ji} + \bar{\pi}_{Ji}}$. The working response y_{ji}^* can be established as

$$y_{ji}^* = \bar{d}_{ji}(y_{ji} - f(\bar{\eta}_{ji})) + \bar{\eta}_{ji} = \eta_{ji} + \bar{d}_{ji} e_{ji}. \tag{S6}$$

Rewrite the model S6 into matrix language

$$\boldsymbol{y}^* = \boldsymbol{D}_\pi (\boldsymbol{y} - \bar{\boldsymbol{\pi}}) + \bar{\boldsymbol{\eta}} = \boldsymbol{\eta} + \boldsymbol{D}_\pi \boldsymbol{e} \tag{S7}$$

where $\boldsymbol{\eta} = \boldsymbol{X\beta} + \boldsymbol{h}$, $\boldsymbol{D}_\pi = diag(\bar{d}_{11}, \ldots, \bar{d}_{1N}, \ldots, \bar{d}_{J-1,1}, \ldots, \bar{d}_{J-1,N})$,
$\boldsymbol{y} = (y_{11}, \ldots, y_{1N}, \ldots, y_{J-1,1}, \ldots, y_{J-1,N})'$, $\boldsymbol{y}^*$, $\boldsymbol{h}$, $\bar{\boldsymbol{\pi}}$, $\bar{\boldsymbol{\eta}}$, $\boldsymbol{\eta}$ and $\boldsymbol{e}$ are stacked in the same way as $\boldsymbol{y}$. $\boldsymbol{y}^*$ marginally has the normal distribution with mean vector $\boldsymbol{X\beta}$ and variance-covariance matrix $\boldsymbol{V} = \boldsymbol{W}^{-1} + \tau\boldsymbol{K}^*$, where $\boldsymbol{X} = \boldsymbol{I}_{J-1} \otimes \begin{bmatrix} 1 & \boldsymbol{x}'_1 \\ \vdots & \vdots \\ 1 & \boldsymbol{x}'_n \end{bmatrix}$, $\boldsymbol{\beta} = (\alpha_1, \boldsymbol{\beta}'_1, \ldots, \alpha_{J-1}, \ldots, \boldsymbol{\beta}'_{J-1})'$, $\boldsymbol{K}^* = \boldsymbol{I}_{J-1} \otimes \boldsymbol{K}$. $\boldsymbol{I}_{J-1}$ is a $(J-1)$-th order identity matrix and $\boldsymbol{K}$ is an $N \times N$ kernel matrix and $\boldsymbol{h} \sim \mathcal{N}(\boldsymbol{0}, \boldsymbol{I}_{J-1} \otimes \tau\boldsymbol{K})$. $\boldsymbol{W} = (\boldsymbol{D}_\pi \boldsymbol{V}_\pi \boldsymbol{D}_\pi)^{-1}$ is the working weight matrix with $\boldsymbol{V}_\pi$ being the variance-covariance matrix evaluated at $\bar{\boldsymbol{\pi}}$. That is, $Var(\boldsymbol{e}|\bar{\boldsymbol{\pi}}) = \boldsymbol{V}_\pi$. The Gaussian log likelihood corresponding to the linear mixed model for $\boldsymbol{y}^*$ is the following:

$$l(\boldsymbol{\beta}, \tau\boldsymbol{K}^*, \boldsymbol{W}; \boldsymbol{y}^*) = -\frac{1}{2}\log|\boldsymbol{V}| - \frac{N \times (J-1)}{2}\log 2\pi - \frac{1}{2}(\boldsymbol{y}^* - \boldsymbol{X\beta})'\boldsymbol{V}^{-1}(\boldsymbol{y}^* - \boldsymbol{X\beta}). \tag{S8}$$

The score function in terms of τ is

$$U(\boldsymbol{\beta}, \tau\boldsymbol{K}^*, \boldsymbol{W}; \boldsymbol{y}^*) = \frac{1}{2}(\boldsymbol{y}^* - \boldsymbol{X\beta})'\boldsymbol{V}^{-1}\boldsymbol{K}^*\boldsymbol{V}^{-1}(\boldsymbol{y}^* - \boldsymbol{X\beta}) - \frac{1}{2}tr|\boldsymbol{V}^{-1}\boldsymbol{K}^*|, \tag{S9}$$

where tr is the trace of matrix. Under the null hypothesis $H_0 : \tau = 0$, $\boldsymbol{V}$ is reduced to $\boldsymbol{W}^{-1}$. Discarding the additive $(tr|\boldsymbol{V}^{-1}\boldsymbol{K}^*|)$ and scaling terms $(\frac{1}{2})$ which will not affect the result of testing, the score test statistic of MiRKAT-MCN for independent data is

$$Q_1 = (\boldsymbol{y}^* - \boldsymbol{X\beta})'\boldsymbol{W}\boldsymbol{K}^*\boldsymbol{W}(\boldsymbol{y}^* - \boldsymbol{X\beta}). \tag{S10}$$

To calculate Q_1, we just need to fit the null model $\boldsymbol{\eta} = \boldsymbol{X\beta}$, and substitute $\boldsymbol{y}^*$, $\boldsymbol{\beta}$ and $\boldsymbol{W}$ with their consistent estimates.

When developing MiRKAT-MCO using the same framework, the assumptions that $\boldsymbol{\beta}_j$ are identical and $\alpha_1 < \cdots < \alpha_{J-1}$ are applied into all the relevant models. We substitute the link function in model S1 with the logit function, and substitute π_{ji}, y_{ji} with the cumulative probability $\nu_{ji} = \sum_{h=1}^{j}\pi_{hi}$ and the cumulative response $\tilde{y}_{ji} = \sum_{h=1}^{j} y_{hi}$, respectively. Hence, $E(e_{ji}|\nu_{ji}) = 0$, $Var(e_{ji}|\nu_{ji}) = \nu_{ji}(1-\nu_{ji})$, and $Cov(e_{ji}, e_{hi}|\nu_{ji}, \nu_{hi}) = \nu_{ji}(1-\nu_{hi})$, $h > j$. $\boldsymbol{D}_\nu$ is the inverse of $\boldsymbol{V}_\nu = Var(\boldsymbol{e}|\boldsymbol{\nu})$.

1.2 MiRKAT-MC for clustered data

The development of MiRKAT-MC for clustered data is fairly similar to that for independent data. We just need to add another random effect to capture the correlation within clusters. Again, we take MiRKAT-MCN for example and the some details of MiRKAT-MCO will be introduced at the end of this section. Let y_{jik}, an indicator denote whether the k-th observation of the i-th cluster belongs to the j-th category, where $k = 1, \dots, m_i$, $i = 1, \dots, n$ and $j = 1, \dots, J$. Let $N = \sum_i m_i$ indicate the total number of observations. Suppose we have the following models

$$\eta_{jik} = \log \frac{\pi_{jik}}{1 - \sum_{j=1}^{J-1} \pi_{jik}} = \alpha_j + \boldsymbol{x}'_{ik}\boldsymbol{\beta}_j + \boldsymbol{u}'_{ik}\boldsymbol{b}_{ji} + h_{jik}, \tag{S11}$$

and

$$y_{jik} = \pi_{jik} + e_{jik}, \tag{S12}$$

where $\pi_{jik} = E(y_{jik}|\boldsymbol{b}_{ji}, h_{jik})$; $\boldsymbol{x}_{ik} = (x_{ik1}, \dots, x_{ikq})'$ denote covariates and α_j and $\boldsymbol{\beta}_j = (\beta_{j1}, \dots, \beta_{jq})'$ are corresponding intercept and coefficients for the fixed effect. We introduce a random variable $\boldsymbol{b}_{ji}$ to measure the correlation between observations within cluster i of category j. $\boldsymbol{u}_{ik}$ are pre-specified coefficients of random effect $\boldsymbol{b}_{ji}$. We consider the model with a random intercept and a random slope in the following derivation. h_{jik} represents the effect of microbiome of k-th observation of cluster i on the category j , with the distribution $\mathcal{N}(0, \tau K_{ik})$. e_{jik} is an unobserved error with $E(e_{jik}|\pi_{jik}) = 0$, $Var(e_{jik}|\pi_{jik}) = \pi_{jik}(1 - \pi_{jik})$ and $Cov(e_{jik}, e_{hik}|\pi_{jik}, \pi_{hik}) = -\pi_{jik}\pi_{hik}$.

We skip steps from 3 to 6 in the above section, and write the model S11 in matrix notation

$$\boldsymbol{\eta} = \boldsymbol{X\beta} + \boldsymbol{Ub} + \boldsymbol{h} \tag{S13}$$

Except for $\boldsymbol{\beta}$ and $\boldsymbol{b}$, each component in the model S13 has three levels - category, cluster, and observation. Thus, for the category level, $\boldsymbol{\eta} = (\boldsymbol{\eta}'_1, \dots, \boldsymbol{\eta}'_{J-1})'$, $\boldsymbol{X} = \boldsymbol{I}_{J-1} \otimes \begin{bmatrix} \mathbf{1}_{m_1} & \boldsymbol{X}_1 \\ \vdots & \vdots \\ \mathbf{1}_{m_n} & \boldsymbol{X}_n \end{bmatrix}$, $\boldsymbol{\beta} = (\alpha_1, \boldsymbol{\beta}'_1, \dots, \alpha_{J-1}, \boldsymbol{\beta}'_{J-1})'$, $\boldsymbol{U} = \boldsymbol{I}_{J-1} \otimes diag(\boldsymbol{U}_1, \dots, \boldsymbol{U}_n)$, $\boldsymbol{b} = (\boldsymbol{b}'_1, \dots, \boldsymbol{b}'_{J-1})'$, $\boldsymbol{h} = (\boldsymbol{h}'_1, \dots, \boldsymbol{h}'_{J-1})'$, where $\mathbf{1}_m$ is $m \times 1$ vector of 1's. For the cluster level, $\boldsymbol{\eta}_j = (\boldsymbol{\eta}'_{j1}, \dots, \boldsymbol{\eta}'_{jn})'$, $\boldsymbol{b}_j = (\boldsymbol{b}'_{j1}, \dots, \boldsymbol{b}'_{jn})'$, $\boldsymbol{h}_j = (\boldsymbol{h}'_{j1}, \dots, \boldsymbol{h}'_{jn})'$. For the observation level, $\boldsymbol{\eta}_{ji} = (\eta_{ji1}, \dots, \eta_{jim_i})'$, $\boldsymbol{X}_i = (\boldsymbol{x}_{i1}, \dots, \boldsymbol{x}_{im_i})'$, $\boldsymbol{U}_i = (\boldsymbol{u}_{i1}, \dots, \boldsymbol{u}_{im_i})'$, $\boldsymbol{h}_{ji} = (h_{ji1}, \dots, h_{jim_i})'$. And $\boldsymbol{x}_{ik} = (x_{ik1}, \dots, x_{ikq})'$, $\boldsymbol{\beta}_j = (\beta_{j1}, \dots, \beta_{jq})'$, $\boldsymbol{u}_{ik} = (1, t_{ik})'$, $\boldsymbol{b}_{ji} = (b_{ji1}, b_{ji2})'$. We assume that $\boldsymbol{b}$ and $\boldsymbol{h}$ are independent, and both of them have the normal distribution, $\boldsymbol{b} \sim \mathcal{N}(\boldsymbol{0}, \boldsymbol{G}^*)$ and $\boldsymbol{h} \sim \mathcal{N}(\boldsymbol{0}, \tau\boldsymbol{K}^*)$. $\boldsymbol{G}^*$ is a $(J-1) \times (J-1)$ block matrix with entries $\boldsymbol{I}_n \otimes \boldsymbol{G}_{jh}$, $j, h = 1, \dots, J-1$, where $\boldsymbol{G}_{jh}$ is a 2×2 matrix, and $\boldsymbol{K}^* = \boldsymbol{I}_{J-1} \otimes \boldsymbol{K}$, where $\boldsymbol{K}$ is an $N \times N$ kernel matrix.

Define the working variable in matrix language as

$$\boldsymbol{y}^* = \boldsymbol{D}_\pi(\boldsymbol{y} - \bar{\boldsymbol{\pi}}) + \bar{\boldsymbol{\eta}} = \boldsymbol{\eta} + \boldsymbol{D}_\pi \boldsymbol{e} \tag{S14}$$

where $\boldsymbol{D}_\pi$ is a diagonal matrix with each entry $\bar{d}_{jik} = \frac{\bar{\pi}_{jik}\bar{\pi}_{jik}}{\bar{\pi}_{jik}+\bar{\pi}_{jik}}$ arranged in the same way as $\boldsymbol{\eta}$. The Gaussian log likelihood corresponding to the linear mixed model for $\boldsymbol{y}^*$ is the following:

$$l(\boldsymbol{\beta}, \boldsymbol{\Sigma}^*; \boldsymbol{y}^*) = -\frac{1}{2}\log|\boldsymbol{\Sigma}^*| - \frac{N \times (J-1)}{2}\log 2\pi - \frac{1}{2}(\boldsymbol{y}^* - \boldsymbol{X\beta})'\boldsymbol{\Sigma}^{*-1}(\boldsymbol{y}^* - \boldsymbol{X\beta}) \tag{S15}$$

where $\boldsymbol{\Sigma}^* = \boldsymbol{W}^{-1} + \tau \boldsymbol{K}^* + \boldsymbol{U}\boldsymbol{G}^*\boldsymbol{U}'$. The score function in terms of τ is

$$U(\boldsymbol{\beta}, \boldsymbol{\Sigma}^*; \boldsymbol{y}^*) = \frac{1}{2}(\boldsymbol{y}^* - \boldsymbol{X\beta})'\boldsymbol{\Sigma}^{*-1}\boldsymbol{K}^*\boldsymbol{\Sigma}^{*-1}(\boldsymbol{y}^* - \boldsymbol{X\beta}) - \frac{1}{2}tr|\boldsymbol{\Sigma}^{*-1}\boldsymbol{K}^*| \tag{S16}$$

Under the null hypothesis $H_0 : \tau = 0$, $\boldsymbol{\Sigma}^*$ is reduced to $\boldsymbol{\Sigma} = \boldsymbol{W}^{-1} + \boldsymbol{U}\boldsymbol{G}^*\boldsymbol{U}'$. Therefore, the score test statistic of MiRKAT-MC for longitudinal data is

$$Q_2 = (\boldsymbol{y}^* - \boldsymbol{X\beta})'\boldsymbol{\Sigma}^{-1}\boldsymbol{K}^*\boldsymbol{\Sigma}^{-1}(\boldsymbol{y}^* - \boldsymbol{X\beta}) \tag{S17}$$

To calculate Q_2, one just needs to fit the null model $\boldsymbol{\eta} = \boldsymbol{X\beta} + \boldsymbol{Ub}$, and substitute $\boldsymbol{y}^*$, $\boldsymbol{\beta}$ and $\boldsymbol{\Sigma}$ with their consistent estimates/predictors.

When developing MiRKAT-MCO using the same framework, the assumptions that $\boldsymbol{\beta}_j$ are identical, $\alpha_1 < \cdots < \alpha_{J-1}$, and $\boldsymbol{b}_{ji}$ are identical across categories for a fixed cluster i are applied to all relevant models. We substitute the link function in model S11 with the logit function, and substitute π_{jik}, y_{jik} with the cumulative probability $\nu_{jik} = \sum_{h=1}^{j} \pi_{hik}$ and the cumulative response $\tilde{y}_{jik} = \sum_{h=1}^{j} y_{hik}$, respectively. Hence, $E(e_{jik}|\nu_{jik}) = 0$, $Var(e_{jik}|\nu_{jik}) = \nu_{jik}(1-\nu_{jik})$, and $Cov(e_{jik}, e_{hik}|\nu_{jik}, \nu_{hik}) = \nu_{jik}(1-\nu_{hik})$, $h > j$. $\boldsymbol{D}_\nu$ is the inverse of $\boldsymbol{V}_\nu = Var(\boldsymbol{e}|\boldsymbol{\nu})$.

2 SUPPLEMENTARY TABLES AND FIGURES

2.1 Tables

Table S1. Empirical type I error rates of MiRKAT-MC for clustered data with a random intercept and a random slope for within-cluster correlation with three category outcomes. n denotes the number of clusters while N denotes the total sample size. g is the variance of random effects. K_w: the weighted UniFrac kernel; K_u: the unweighted UniFrac kernel; K_{BC}: the Bray-Curtis kernel; K_5: the generalized UniFrac kernel with parameter 0.5; HMP: the omnibus test using harmonic mean *p* value test.

	$n = 30$ ($N = 105$)			$n = 60$ ($N = 210$)		
g	0.25	1	4	0.25	1	4
MiRKAT-MCN						
K_w	0.0470	0.0476	0.0441	0.0464	0.0472	0.0464
K_u	0.0538	0.0519	0.0501	0.0525	0.0515	0.0516
K_{BC}	0.0478	0.0453	0.0503	0.0497	0.0462	0.0497
K_5	0.0494	0.0494	0.0506	0.0486	0.0484	0.0452
HMP	0.0478	0.0456	0.0483	0.0479	0.0480	0.0462
MiRKAT-MCO						
K_w	0.0444	0.0443	0.0523	0.0492	0.0439	0.0445
K_u	0.0528	0.0524	0.0498	0.0494	0.0501	0.0463
K_{BC}	0.0493	0.0503	0.0501	0.0513	0.0504	0.0477
K_5	0.0491	0.0484	0.0548	0.0484	0.0507	0.0494
HMP	0.0473	0.0466	0.0463	0.0481	0.0476	0.0475

2.2 Figures

Table S2. Empirical type I error rates of MiRKAT-MC for clustered data with a random intercept and a random slope for within-cluster correlation with three-category outcomes. n indicates the number of clusters while N is the number of total observations. g denotes the variance of random effects. The definition of K_w, K_u, K_{BC}, K_5 and HMP is the same as Table S1.

	$n = 30$ ($N = 105$)			$n = 60$ ($N = 210$)		
g	0.25	1	4	0.25	1	4
MiRKAT-MCN						
K_w	0.0498	0.0492	0.0467	0.0478	0.0496	0.0484
K_u	0.0521	0.0533	0.0486	0.0449	0.0508	0.0478
K_{BC}	0.0519	0.0542	0.0494	0.0522	0.0478	0.0497
K_5	0.0527	0.0516	0.0521	0.0521	0.0468	0.0505
HMP	0.0514	0.0533	0.0472	0.0465	0.0478	0.0488
MiRKAT-MCO						
K_w	0.0500	0.0473	0.0474	0.0449	0.0498	0.0457
K_u	0.0486	0.0506	0.0487	0.0483	0.0483	0.0538
K_{BC}	0.0535	0.0507	0.0487	0.0453	0.0493	0.0485
K_5	0.0519	0.0471	0.0489	0.0476	0.0501	0.0486
HMP	0.0495	0.0467	0.0481	0.0452	0.0483	0.0475

Table S3. Empirical type I error rates of MiRKAT-MC for clustered data with a random intercept with five-category outcomes. n denotes the number of clusters while N denotes the total sample size. g is the variance of random effects. The definition of K_w, K_u, K_{BC}, K_5 and HMP is the same as Table S1.

	$n = 50$ ($N = 175$)			$n = 100$ ($N = 350$)		
g	0.25	1	4	0.25	1	4
MiRKAT-MCN						
K_w	0.0495	0.0487	0.0480	0.0416	0.0417	0.0454
K_u	0.0473	0.0510	0.0519	0.0542	0.0470	0.0501
K_{BC}	0.0524	0.0490	0.0521	0.0462	0.0454	0.0469
K_5	0.0528	0.0491	0.0499	0.0476	0.0495	0.0513
HMP	0.0492	0.0490	0.0469	0.0450	0.0435	0.0483
MiRKAT-MCO						
K_w	0.0439	0.0450	0.0481	0.0430	0.0412	0.0466
K_u	0.0481	0.0525	0.0479	0.0505	0.0541	0.0522
K_{BC}	0.0460	0.0474	0.0477	0.0494	0.0455	0.0499
K_5	0.0517	0.0500	0.0496	0.0471	0.0450	0.0517
HMP	0.0460	0.0500	0.0463	0.0438	0.0459	0.0480

Table S4. Empirical type I error rates of MiRKAT-MC for clustered data with a random intercept and a random slope with five-category outcomes. n denotes the number of clusters while N denotes the total sample size. g is the variance of random effects. The definition of K_w, K_u, K_{BC}, K_5 and HMP is the same as Table S1.

	$n = 50$ ($N = 175$)			$n = 100$ ($N = 350$)		
g	0.25	1	4	0.25	1	4
MiRKAT-MCN						
K_w	0.0489	0.0491	0.0472	0.0480	0.0458	0.0494
K_u	0.0489	0.0503	0.0460	0.0493	0.0515	0.0482
K_{BC}	0.0452	0.0419	0.0420	0.0496	0.0511	0.0484
K_5	0.0492	0.0490	0.0525	0.0469	0.0488	0.0507
HMP	0.0487	0.0452	0.0446	0.0467	0.0457	0.0483
MiRKAT-MCO						
K_w	0.0477	0.0449	0.0436	0.0480	0.0481	0.0478
K_u	0.0495	0.0513	0.0530	0.0480	0.0507	0.0524
K_{BC}	0.0471	0.0486	0.0494	0.0491	0.0518	0.0471
K_5	0.0521	0.0523	0.0464	0.0465	0.0528	0.0527
HMP	0.0452	0.0509	0.0436	0.0489	0.0516	0.0475

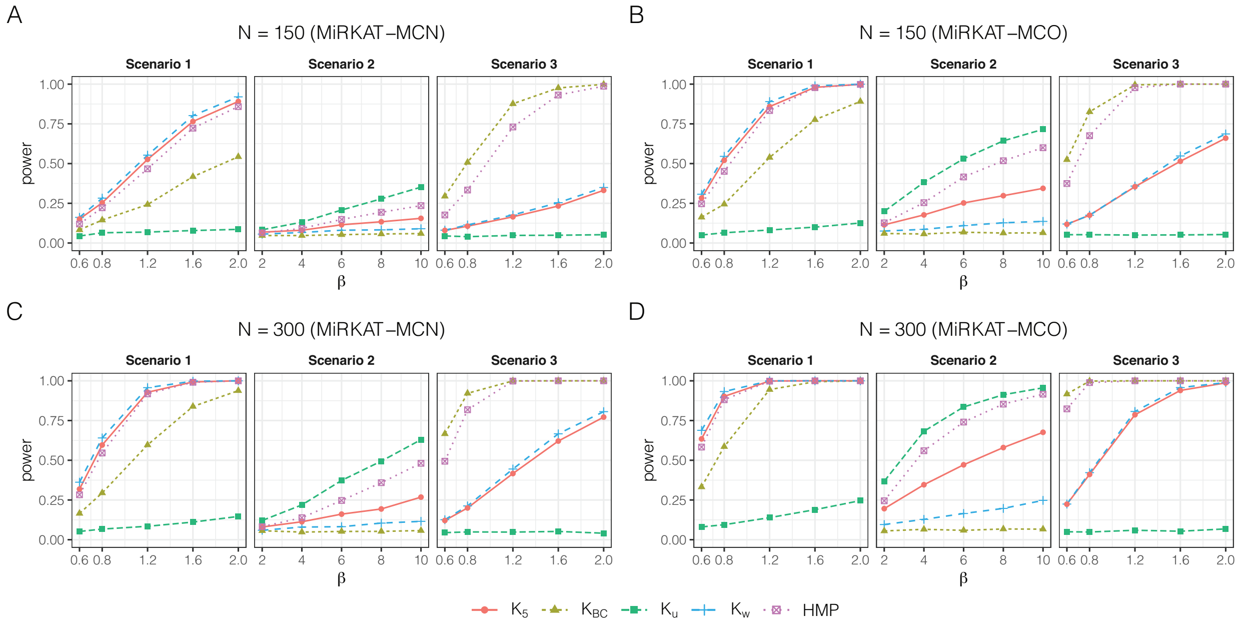

Figure S1. Statistical powers of MiRKAT-MC for independent data with five categories. Scenario 1: $\mathcal{A}$={A random selected common cluster among 20 clusters by PAM}; Scenario 2: $\mathcal{A}$ = {The rarest cluster among 20 clusters by PAM}; Scenario 3: $\mathcal{A}$ = {10 most abundant OTUs}. K_w: the weighted UniFrac kernel; K_u: the unweighted UniFrac kernel; K_{BC}: the Bray-Curtis kernel; K_5: the generalized UniFrac kernel with parameter 0.5; HMP: the omnibus test using harmonic mean p value test. (A) MiRKAT-MCN with 150 total samples; (B) MiRKAT-MCO with 150 total samples; (C) MiRKAT-MCN with 300 total samples; (D) MiRKAT-MCO with 300 total samples.

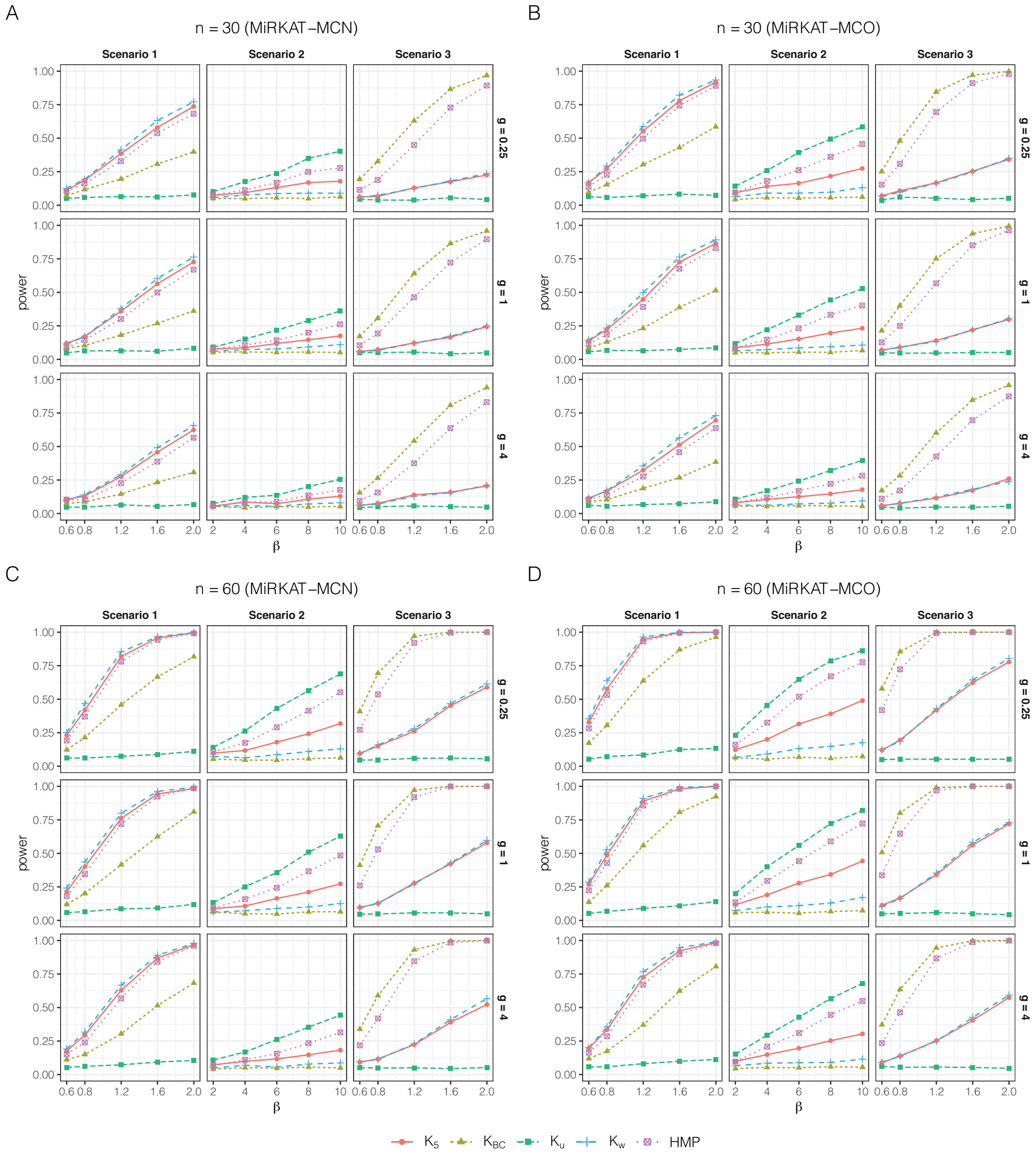

Figure S2. Statistical powers of MiRKAT-MC for clustered data with a random intercept model with three-category data. g denotes the variance of random effects. The definition of scenarios and K_w, K_u, K_{BC}, K_5 and HMP are the same as Figure S1. (A) MiRKAT-MCN with 30 clusters (105 total samples); (B) MiRKAT-MCO with 30 clusters (105 total samples); (C) MiRKAT-MCN with 60 clusters (210 total samples); (D) MiRKAT-MCO with 60 clusters (210 total samples).

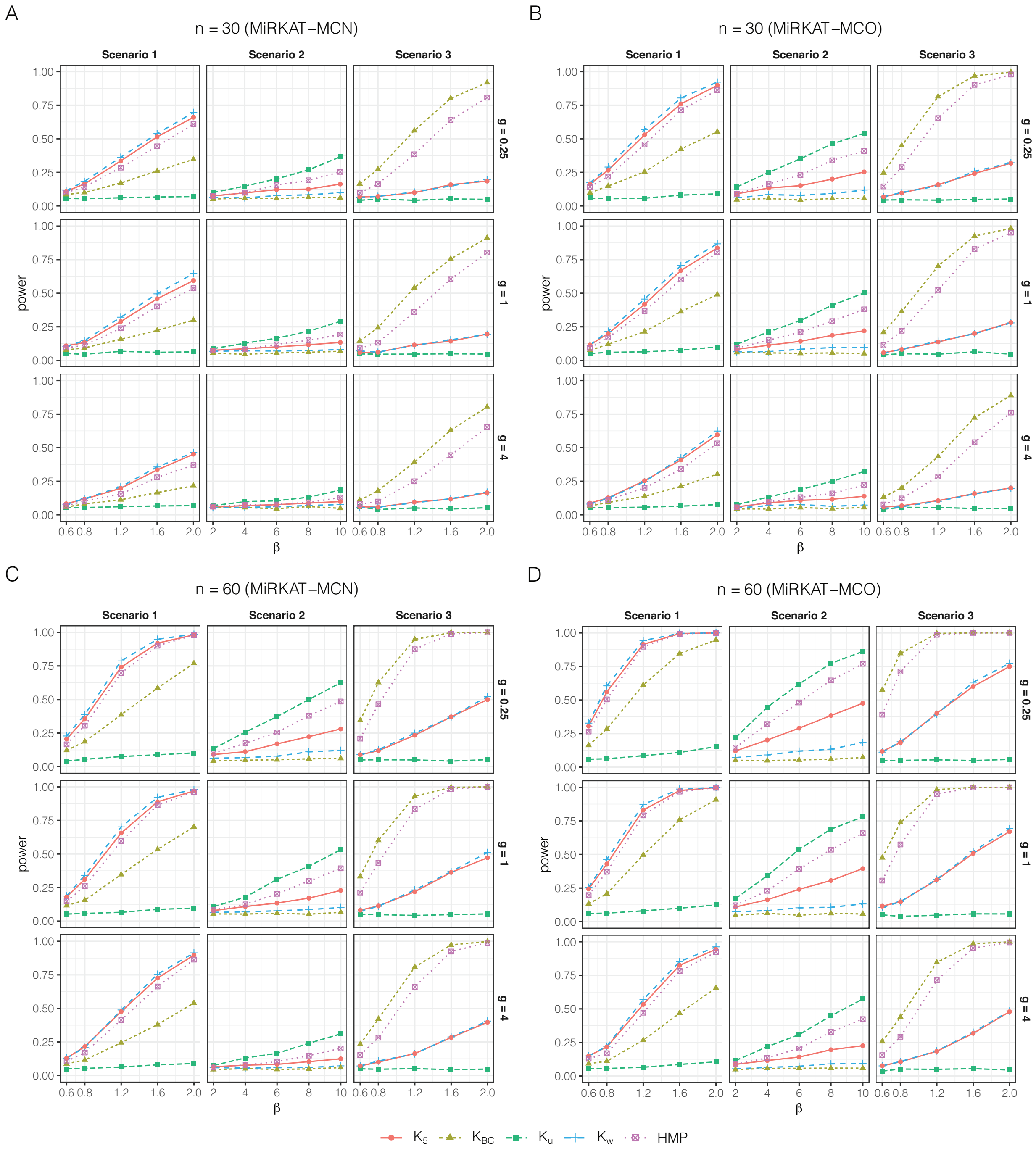

Figure S3. Statistical powers of MiRKAT-MC for clustered data with a random intercept and a random slope model with three-category data. g denotes the variance of random effects. The definition of K_w, K_u, K_{BC}, K_5 and HMP is the same as Figure S1. (A) MiRKAT-MCN with 30 clusters (105 total samples); (B) MiRKAT-MCO with 30 clusters (105 total samples); (C) MiRKAT-MCN with 60 clusters (210 total samples); (D) MiRKAT-MCO with 60 clusters (210 total samples).

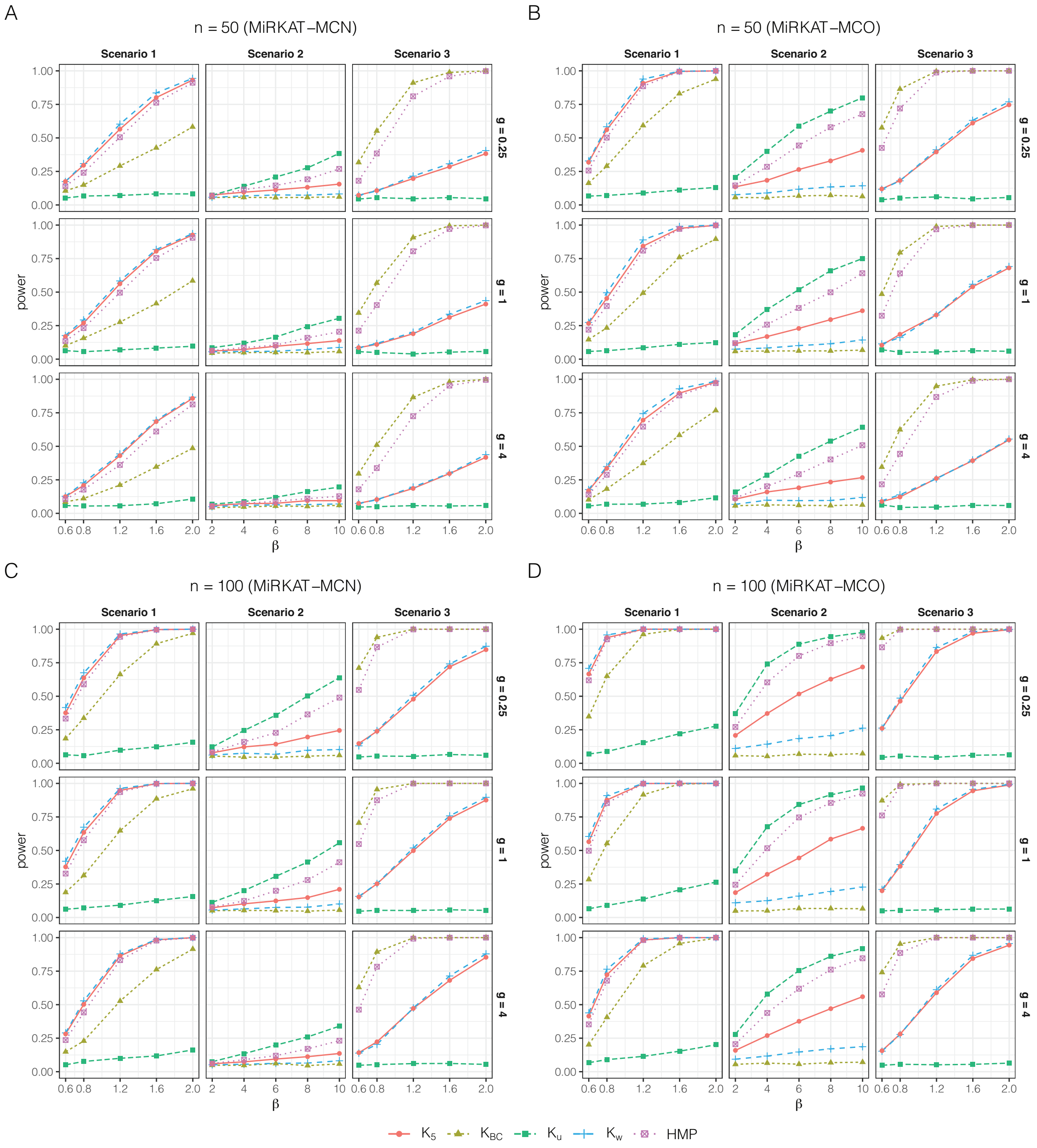

Figure S4. Statistical powers of MiRKAT-MC for clustered data with a random intercept model with five-category data. g denotes the variance of random effects. The definition of scenarios and K_w, K_u, K_{BC}, K_5 and HMP are the same as Figure S1. (A) MiRKAT-MCN with 50 clusters (175 total samples); (B) MiRKAT-MCO with 50 clusters (175 total samples); (C) MiRKAT-MCN with 100 clusters (350 total samples); (D) MiRKAT-MCO with 100 clusters (350 total samples).

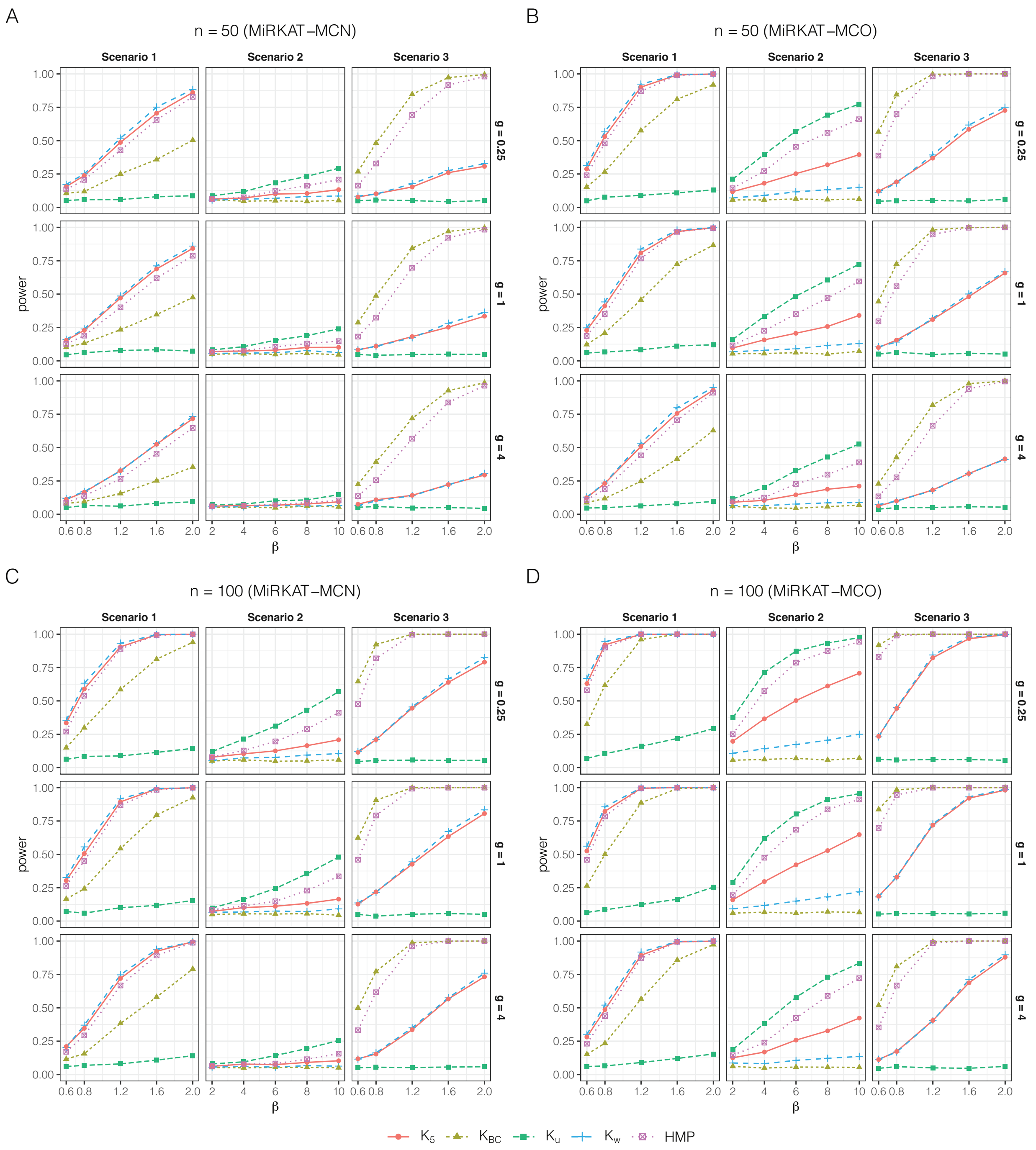

Figure S5. Statistical powers of MiRKAT-MC for clustered data with a random intercepts and a random slope model with five-category data. g denotes the variance of random effects. The definition of scenarios and K_w, K_u, K_{BC}, K_5 and HMP are the same as Figure S1. (A) MiRKAT-MCN with 50 clusters (175 total samples); (B) MiRKAT-MCO with 50 clusters (175 total samples); (C) MiRKAT-MCN with 100 clusters (350 total samples); (D) MiRKAT-MCO with 100 clusters (350 total samples).
